# Conformational Stability of the NH_2_-Terminal Propeptide of the Precursor of Pulmonary Surfactant Protein SP-B

**DOI:** 10.1371/journal.pone.0158430

**Published:** 2016-07-05

**Authors:** Ángeles Bañares-Hidalgo, Jesús Pérez-Gil, Pilar Estrada

**Affiliations:** Departamento de Bioquímica y Biología Molecular I, Facultad de Biología, Universidad Complutense, Ciudad Universitaria, 28040, Madrid, Spain; CNR, ITALY

## Abstract

Assembly of pulmonary surfactant lipid-protein complexes depends on conformational changes coupled with proteolytic maturation of proSP-B, the precursor of pulmonary surfactant protein B (SP-B), along the surfactant biogenesis pathway in pneumocytes. Conformational destabilization of the N-terminal propeptide of proSP-B (SP-B_N_) triggers exposure of the mature SP-B domain for insertion into surfactant lipids. We have studied the conformational stability during GdmCl- or urea-promoted unfolding of SP-B_N_ with trp fluorescence and circular dichroism spectroscopies. Binding of the intermediate states to bis-ANS suggests their molten globule-like character. ΔG^0^_H2O_ was ~ 12.7 kJ·mol^-1^ either with urea or GdmCl. None of the thermal transitions of SP-B_N_ detected by CD correspond to protein unfolding. Differential scanning calorimetry of SP-B_N_ evidenced two endothermic peaks involved in oligomer dissociation as confirmed with 2 M urea. Ionic strength was relevant since at 150 mM NaCl, the process originating the endotherm at the highest temperature was irreversible (T_m2_ = 108.5°C) with an activation energy of 703.8 kJ·mol^-1^. At 500 mM NaCl the process became reversible (T_m2_ = 114.4°C) and data were fitted to the Non-two States model with two subpeaks. No free thiols in the propeptide could be titrated by DTNB with or without 5.7 M GdmCl, indicating disulfide bonds establishment.

## Introduction

The function of a protein depends on its ability to adopt a specific structure. Proteins can form partially folded, collapsed states resembling the intermediate states along the protein folding pathway, and this is important in understanding the mechanisms of protein folding [1]. Those states have been often considered as molten globule structures, that is, partially folded proteins with native-like secondary structure, but lacking the extensive, specific side-chain packing interactions of the native structure [2]. In addition to the molten globule state, two kinds of intermediate states called the pre-molten globule state and the highly-ordered molten globule state may exist during protein folding and refolding [3]. The protein in the pre-molten globule state is half-way between the molten globule and the unfolded state, it is less compact than the molten globule but is still more compact than the random coil [4] whereas the highly-ordered molten globule has more structure and is much more similar to the native state [5]. Intermediate states in the folding-unfolding processes may be revealed by mild denaturing conditions, such as changes in pressure, temperature, pH or upon addition of chaotropes (i.e. urea or guanidinium chloride) [6].

We are interested in the study of proteins involved in pulmonary surfactant biogenesis and function from a structural and functional point of view. The stability of the respiratory surface at the lung is maintained by lipids and proteins synthesized and secreted by type II pneumocytes into the alveolar spaces, to form a film at the air-water interface known as pulmonary surfactant. The surfactant protein B (SP-B) is strictly necessary to promote the formation of this film in order to prevent collapse during deflation [7] and is produced from a precursor (proSP-B) containing two propeptides flanking the mature protein at its NH_2_- and COOH-termini respectively (S1 Fig). The 177 amino acid propeptide flanking the NH_2_ terminus of the mature protein is necessary and sufficient for targeting processing and assembly of the hydrophobic mature SP-B into surfactant complexes *in vivo* [8]. Since deletion of this propeptide results in accumulation of SP-B within the endoplasmic reticulum, a role as intramolecular chaperone for SP-B has been suggested [9]. Moreover, proSP-B is an homologous protein to prosaposin, the precursor protein of four saposins (*sphingolipid activator proteins*) generated through cleavage of the precursor prosaposin [10]. Prosaposin contains four Saposin B (SAPB) domains and two Saposin A (SAPA) modules, one at its NH_2_ and another at its COOH terminus whereas proSP-B contains three SAPB modules (in mature SP-B and in both flanking propeptides) plus an additional SAPA module in the NH_2_-terminal propeptide (S1 Fig). Other members of the saposin-like family proteins (SAPLIPs), such as NK-lysin or the amoebapore have antipathogenic activities [11]. As occurs with prosaposin, it has been proposed that the propeptides of proSP-B could have additional functions in the lung once released from the mature protein. This has been confirmed after isolation of the saposin module from the NH_2_-terminal propeptide of proSP-B from bronchoalveolar lavage fluid and its characterization as participating in the host defence in rat lung [12]. The structural features of the NH_2_-terminal propeptide of proSP-B (SP-B_N_) are therefore important not only to understand SP-B and surfactant biogenesis but also to unravel the intrinsic structure-function determinants of a protein that may have its own role to protect the pulmonary surface from the entrance of pathogens.

In this context, we have produced a recombinant form of human SP-B_N_, soluble and containing the microbicide module [13] and the conditions that favoured or hindered its aggregation have been studied [14]. Also we have observed that acidic pH triggers, in the propeptide, the formation of a coiled coil structure [15]. In the present work, we extend the structural dissection of the protein by analyzing the thermodynamical stability of the propeptide, and the detection of their folding intermediates, if any, in the presence of chaotropes. Results obtained employing far-UV circular dichroism, intrinsic and extrinsic fluorescence spectroscopy and differential scanning calorimetry are discussed regarding the conformational stability of the protein and its oligomeric state determined through sedimentation velocity. The lack of free cysteines in the recombinant protein indicates that disulfide bonds have been formed in the propeptide. This is relevant since proteins of the SAPLIP family contain conserved cysteines, which are involved in intramolecular disulfide bonds responsible for their high thermal stability and resistance to proteolytic degradation [16, 17]. Still, our results reveal a structural complexity in SP-B_N_ that should be taken into account to understand the mechanisms triggering the liberation and assembly of surface active and antipathogenic modules.

## Materials and Methods

### Production and Purification of SP-B_N_

The human wild-type propeptide was expressed in *E*. *coli* as a fusion protein (MBP-SP-B_N_) with the Maltose Binding Protein (MBP) and purified after cleaving the fusion with factor Xa as described [13]. The fractions containing the purified SP-B_N_ in 20 mM Tris-HCl buffer pH 7, 500 mM NaCl were dialyzed towards the buffer needed in subsequent experiments. The mean neat charge of the propeptide is defined as the net charge at pH 7 [18] divided by the total number of residues. The hydrophobicity at each sequence position was calculated by the Kite and Doolittle scale [19] using a window size of 5 amino acids and normalized to a scale of 0–1. The mean hydrophobicity is defined as the sum of the normalized hidrophobicities of all residues divided by the number of residues of the propeptide.

### Far-UV Circular Dichroism Experiments

Circular dichroism spectra of SP-B_N_ were recorded at 25°C in a Jasco J-715 spectropolarimeter using thermostated quartz cells of 0.1-cm path length, at 50 nm·min^-1^ (1 s response time) for the far-UV (250–195 nm) spectral range, each spectrum being the accumulation of 5 scans. The spectra were obtained in 200 μL of 5mM acetate, 5 mM MES, 5 mM Tris-HCl buffer (AMT buffer) 150 mM NaCl pH 7 at 0.115 mg·mL^-1^ protein. Mean residue molar ellipticities [θ] were calculated from the measured ellipticity taking into account the protein concentration, the molecular weight of SP-B_N_ (19,902 Da, DNAstar program) and the number of amino acids per molecule (177). Estimations of the secondary structure content from the CD spectra were performed by using the CDPro suite program and the α-helix and β-sheet contents were calculated using three different methods, CONTIN/LL, SELCON3 and CDSSTR employing their mean value [20]. CD spectra in the presence of chaotropes were obtained after preincubating the samples with the denaturant for 1 h at 25°C. Samples at the corresponding chaotrope concentration, 0.8–8 M GdmCl and 0.5–7.5 M urea, were prepared from stock solutions of 9.75 M GdmCl (Sigma) and 9.87 M urea (Sigma) respectively. Control samples in the absence of protein were used to subtract a baseline from samples with protein. The reversibility of chemical unfolding was analyzed by dialyzing extensively samples with the highest chaotrope concentration towards 5 mM AMT buffer, 150 mM NaCl pH 7 at 4°C and by recording the spectra thereafter.

Temperature studies were carried out with 0.115 mg·mL^-1^ protein in 5 mM AMT buffer pH 7 with 150 or 500 mM NaCl. The temperature dependence of the CD signal was determined by heating the samples from 25 to 85°C at 30°C·h^-1^ and collecting the ellipticity at 208 nm every 0.2°C. After the T ramp the sample was cooled back to 25°C and the CD spectrum was recorded again.

### Intrinsic and Extrinsic Fluorescence Studies

The intrinsic fluorescence emission spectra of the propeptide were recorded at 25°C in a SLM-Aminco AB2 spectrofluorimeter using a 1-cm quartz cell with excitation (290 nm) and emission slits set at 4 nm and scan speed of 2 nm·s^-1^. Samples contained 0.09 mg·mL^-1^ protein in 5 mM AMT buffer, 150 mM NaCl pH 7. The spectra of the protein in the presence of chaotropes, 0.8–6.6 M GdmCl or 0.5–7.5 M urea were recorded after preincubating the samples for 4 h at 25°C. Spectra were also recorded of samples dialyzed as described above to check the reversibility of protein unfolding.

Extrinsic fluorescence of 11 μM bis-ANS probe (4,4´-bis-1-phenylamine-8-naphftalene sulfonate from Thermo Fisher Scientific) was determined as follows. Samples containing chaotropes were prepared in a final volume of 200 μL by adding 11 μL of the probe from a 200 μM solution in methanol to 29 μg protein in 5 mM AMT buffer, 150 mM NaCl pH 7 with the corresponding amount of GdmCl or urea. After incubation at 37°C for 5 min, the emission spectra (400–625 nm) were recorded in the spectrofluorimeter connected to a water-bath thermostatized at 37°C. The excitation wavelength was 395 nm and samples without protein were used as blank. Scan speed and slit widths were as described above.

### Analysis of CD and Fluorescence Emission Data

The transition curves obtained by CD and fluorescence spectroscopy from the propeptide unfolding experiments were analyzed according to the following equations:

$$Y_{obs}=\frac{Y_{N}+Y_{U}\text{exp}\left(\frac{-\Delta G_{H2O}^{0}+mD}{RT}\right)}{1+\text{exp}\left(\frac{-\Delta G_{H2O}^{0}+mD}{RT}\right)}$$ (1)

$$Y_{obs}=\frac{Y_{N}+a}{1+\text{exp}\left(\frac{D_{1/2}-D}{b}\right)}$$ (2)

$$\Delta G^{0}=-RT\text{ln}\left(\frac{Y_{N}-Y_{obs}}{Y_{obs}-Y_{U}}\right)$$ (3)

$$\Delta G^{0}=\Delta H^{0}-T\Delta S^{0}$$ (4)

In the unfolding process by chaotropes, in Eqs (1) and (2): Y_obs_ is the observed parameter ([θ]^220^ or FI_350_) at each denaturant concentration, Y_N_ and Y_U_ are the parameter values in native and unfolded conditions respectively, D is the denaturant concentration (mol·L^-1^), ΔG^0^_H2O_ is the change of free energy in the absence of denaturant (kJ·mol^-1^), *m* is a measure of the dependence of the free energy on the denaturant concentration (kJ·mol^-1^·M^-1^), R is the gas constant (8.314 J·mol^-1^·K^-1^) and T is the absolute temperature (K). The midpoint of the unfolding curve (D_1/2_) is the denaturant concentration at which half of the protein is unfolded and can be calculated as D_1/2_ = ΔG^0^_H2O_/*m* since ΔG^0^_H2O_ and *m* are obtained from Eq (1). Alternatively, D_1/2_ can be determined directly from fitting the same data to Eq (2) being *b* and *a* constants. In temperature analysis, D and D_1/2_ in Eq (2) were substituted by T and the transition temperatures respectively (all of them in°C for easy recognition) and Y_obs_ was [θ]^208^ in Eqs (2) and (3). The change of free energy, ΔG^0^ was calculated for each temperature (K) according to Eq (3). The enthalpy change, ΔH^0^ (kJ·mol^-1^) and the entropy change, ΔS^0^ (kJ·mol^-1^·K^-1^) were the intercept and the slope, respectively, in Eq (4)

### Analytical Ultracentrifugation

Hydrodynamic studies of 0.15 mg·ml^-1^ SP-B_N_ in 5 mM Tris-HCl pH 7.0 containing either, 0, 150 or 500 mM NaCl were performed at 20°C and 48,000 rpm in an Optima XL-1 (Beckman-Coulter Inc) analytical ultracentrifuge equipped with UV-visible optics. The partial specific volume $\bar{V}$ of SP-B_N_ was 0.73 mL·g^-1^ estimated from its amino acid composition with the program SEDNTERP, version 1.09 (retrieved from RASMB server) [21]; the solvent density ρ was 1.000 g·mL^-1^ and the solvent viscosity η was 1.002 cpoise in the absence of salt, 1.005 g·mL^-1^ and 1.017 cpoise with 150 mM NaCl and 1.019 g·mL^-1^ and 1.049 cpoise with 500 mM NaCl respectively, estimated with the same program.

### Differential Scanning Calorimetry

Differential scanning calorimetry (DSC) experiments were performed on a VP-DSC (MicroCal) differential scanning microcalorimeter with a cell volume of 514.9 μL. The propeptide (0.2–0.6 mg·mL^-1^) in 5 mM Tris-HCl buffer pH 7, 150 or 500 mM NaCl and the corresponding buffer were degassed for 3 min at room temperature in a chamber under vacuum and gentle stirring and then loaded into the sample and reference cells where overpressure was kept to prevent degassing. The measurements were taken every 0.1°C and the scan rate was 60°C·h^-1^. DSC scans began at 20°C and were over at the highest temperature possible in the VP-DSC (~ 120°C). Second scans were obtained by reheating samples after cooling them for 20 min upon the completion of the first scan. The apparent Cp profiles were obtained by subtracting the instrumental baseline (obtained with buffer in both cells) from the experimental thermograms. Then, the thermograms were normalized for protein concentration based on a monomer of 19,902 Da and the pre- and post-transition baselines were subtracted.

The reversible transitions were subjected to thermodynamic deconvolution analysis and the thermogram peak of each successive scan was fitted to the Non Two-State model with two peaks according to Eq (5) of the DSC tutorial guide with the Origin Microcal software, as this model accounted for the best fitting of the data (the lowest χ^2^ / DoF (Degree of Freedom)).

$$Cp(T)=\frac{\text{exp}\left\{\frac{-\Delta H_{VH1}}{RT}\left(1-\frac{T}{T_{m1}}\right)\right\}\Delta H_{VH1}\Delta H_{cal1}{}^{{}_{}}}{{\left[1+\text{exp}\left\{\frac{-\Delta H_{VH1}}{RT}\left(1-\frac{T}{T_{m1}}\right)\right\}\right]}^{2}RT^{2}}+\frac{\text{exp}\left\{\frac{-\Delta H_{VH2}}{RT}\left(1-\frac{T}{T_{m2}}\right)\right\}\Delta H_{VH2}\Delta H_{cal2}{{}_{}}^{{}_{}}}{{\left[1+\text{exp}\left\{\frac{-\Delta H_{VH2}}{RT}\left(1-\frac{T}{T_{m2}}\right)\right\}\right]}^{2}RT^{2}}$$ (5)

where T_m1_ (melting temperature), ΔH_VH1_ (van´t Hoff enthalpy change) and ΔH_cal1_ (calorific enthalpy change) account for the thermal transition of domain 1 whereas T_m2_, ΔH_VH2_ and ΔH_cal2_ account for the thermal transition of domain 2. Cp is the excess heat capacity (kJ·mol^-1^·K^-1^).

The activation energy for irreversible transitions (EA), considered as one-step processes from native to irreversibly inactivated state of the protein [22], was determined by Eq (6) where Cp^ex^_max_ is the maximum excess of heat capacity obtained at the T_m_.

$$E_{A}=\frac{eRT_{m}^{2}Cp_{\text{max}}^{ex}}{\Delta H_{cal}}$$ (6)

Samples of 0.6 mg·mL^-1^protein in 5 mM Tris-ClH buffer 150 mM NaCl pH 7 containing 2 M urea in the same buffer were subjected to DSC in the temperature range 20–120°C at 60°C·h^-1^ scan rate. The chaotrope was added to the reference cell at the same concentration than was added to the sample cell.

### Analytical Procedures

Along the propeptide production and purification processes, protein concentration was routinely determined with colorimetric methods as described [13] but once purified, the propeptide concentration was calculated from its absorbance at 280 nm using 20,790 M^-1^·cm^-1^ as molar extintion coefficient at 280 nm [14].

Free thiols of purified SP-B_N_ were titrated by adding 40 μL of 5 mM 5,5´- dithio-bis-(2-nitrobenzoic acid) (DTNB, Sigma) to 38.4 μg of protein in 960 μL of 5 mM Tris-HCl pH 8.2, 150 mM NaCl (assay buffer). Protein was previously dialyzed towards the same buffer at 4°C. The absorbance at 412 nm was recorded after 2 h at 22°C to check the release of 5-thiobis-(2-nitrobenzoic acid) (TNB) [23]. L-Cysteine (Fluka) was the standard and ε_412_ = 13,800 M^-1^cm^-1^ was used to quantify cysteines. For titration under denaturing conditions the same protocol was applied except that the protein was preincubated with the assay buffer containing 5.7 M GdmCl, which was added 30 min prior to DTNB addition. Free thiols were also analyzed in the fusion MBP-SP-B_N_ (33.5 μg) and in MBP (75.2 μg; released from the fusion by factor Xa) after removing the 2-mercaptoethanol contained in the purification buffer by extensive dialysis towards the assay buffer.

## Results

### Effect of Chaotropes on the Secondary Structure of SP-B_N_

**Fig 1:**
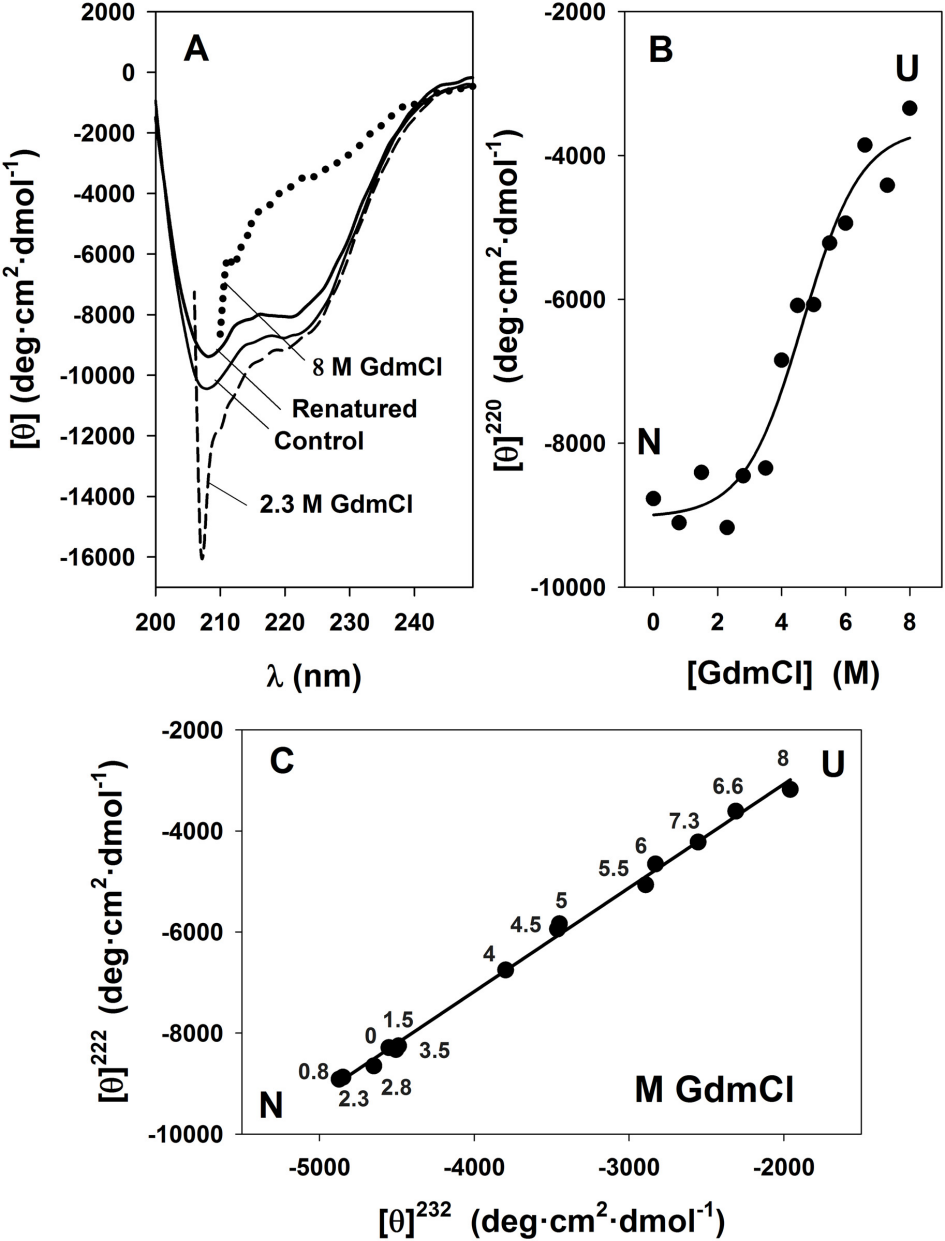
Effect of GdmCl on the secondary structure of SP-B_N_ measured by far-UV circular dichroism spectroscopy. (A) Spectra of the propeptide recorded in 5 mM AMT buffer, 150 mM NaCl pH 7 (control, solid line) and with 2.3 M GdmCl (dashed line), 8 M GdmCl (dotted line) or 8 M GdmCl and ulterior dialysis (renatured, solid line). Spectra recorded at other GdmCl concentrations are not depicted for clarity. (B) Variation of the mean residue ellipticity at 220 nm with 0–8 M GdmCl. The line is a fit of the experimental data to Eq (1) (r = 0.983). N and U are native and unfolded forms of the propeptide. (C) Phase diagram of 0–8 M GdmCl based on far-UV CD (mean residue molar ellipticity at 222 nm *vs* 232 nm). The line is the lineal regression fit of the data (r = 0.99). Numbers beside the points are denaturant concentration.

The chemical unfolding of the propeptide was achieved with GdmCl and urea. The far-UV CD spectra of the propeptide in the presence of 0–8 M GdmCl are depicted in Fig 1.

GdmCl is an electrolyte, and thus it is able to establish undesirable protein-denaturant electrostatic interactions [24] but the ionic strength provided by the salt (150 mM NaCl) in the sample must screen the charges on the surface of the protein, avoiding those interactions. Fig 1A shows the propeptide spectrum recorded in the absence of denaturant (control, solid line) and the spectra obtained in the presence of 2.3 M GdmCl (dashed line) and 8 M GdmCl (dotted line). The shape of the spectrum in the presence of 8 M GdmCl indicates that the protein is unfolded. To check the reversibility of the unfolding process, the chaotrope was eliminated from the sample containing 8 M GdmCl through exhaustive dialysis at 4°C towards the buffer without denaturant, and the spectrum was recorded again (renatured, solid line). As the spectra of control and renatured sample are quite close (92% of the control molar ellipticity was recovered), we can assume that renaturation is achieved, and this allows the thermodynamic treatment of the data. The unfolding transition in Fig 1B shows the variation of the molar ellipticity at 220 nm ([θ]^220^) as [GdmCl] is increased. The data were fitted to Eq (1) and the sigmoid indicates that the backbone of the propeptide unfolds cooperatively, following a two-state transition process from the native protein (N) to (U), the assumed unfolded state, since no clear plateau was reached. No accumulation of intermediate states was detected in the unfolding process. The thermodynamic parameters obtained through the fitting, ΔG^0^_H2O_ = 12.7±3.2 kJ·mol^-1^ and *m* = 2.7±0.7 kJ·mol^-1^·M^-1^, are summarized in Table 1.

**Table 1:** Thermodynamic parameters of SP-B_N_ unfolding by GdmCl and urea followed by tryptophan fluorescence and far-UV circular dichroism.

	[θ]^220^			FI_350_		
	D_1/2_ (M)	ΔG^0^_H2O_ (kJ·mol^-1^)	*m* (kJ·mol^-1^·M^-1^)	D_1/2_ (M)	ΔG^0^_H2O_ (kJ·mol^-1^)	*m* (kJ·mol^-1^·M^-1^)
**GdmCl**	4.7±0.2 (N-U)	12.7±3.2 (N-U)	2.7±0.7 (N-U)	3.8±0.2 (I_1_-U)	12.8±3.3 (I_1_-U)	3.4±0.8 (I_1_-U)
**Urea**	5.2±0.3 (I-U)	12.8±3.1 (I-U)	2.5±0.7 (I-U)	ND	ND	ND

**Fig 2:**
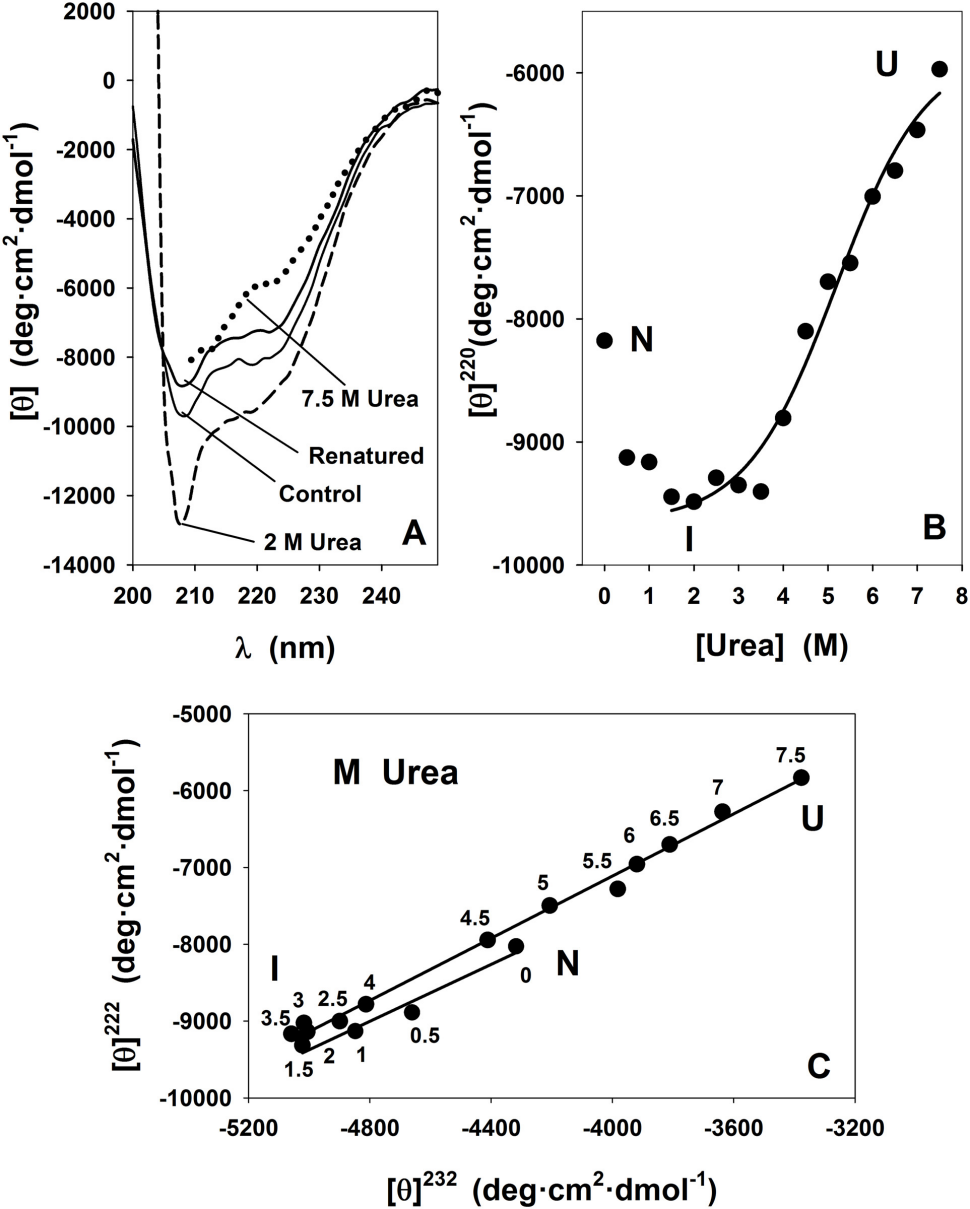
Effect of Urea on the secondary structure of SP-B_N_ measured by far-UV circular dichroism spectroscopy. (A) Spectra of the propeptide recorded in 5 mM AMT buffer, 150 mM NaCl pH 7 (control, solid line), with 2 M urea (dashed line), with 7.5 M urea (dotted line) and with 7.5 M urea and ulterior dialysis (renatured, solid line). (B) Mean residue molar ellipticity at 220 nm *vs* urea concentration. The line is a fit of part of the data (1.5–7.5 M urea; r = 0.99) to Eq (1). (C) Phase diagram analysis of urea-induced equilibrium unfolding of SP-B_N_. The lines (0–1.5 M; r = 0.98) and (1.5–7.5 M; r = 0.99) were obtained by linear regression. I is an intermediate state in the propeptide unfolding process; N and U as in Fig 1. Numbers beside the points are denaturant concentration.

**Fig 3:**
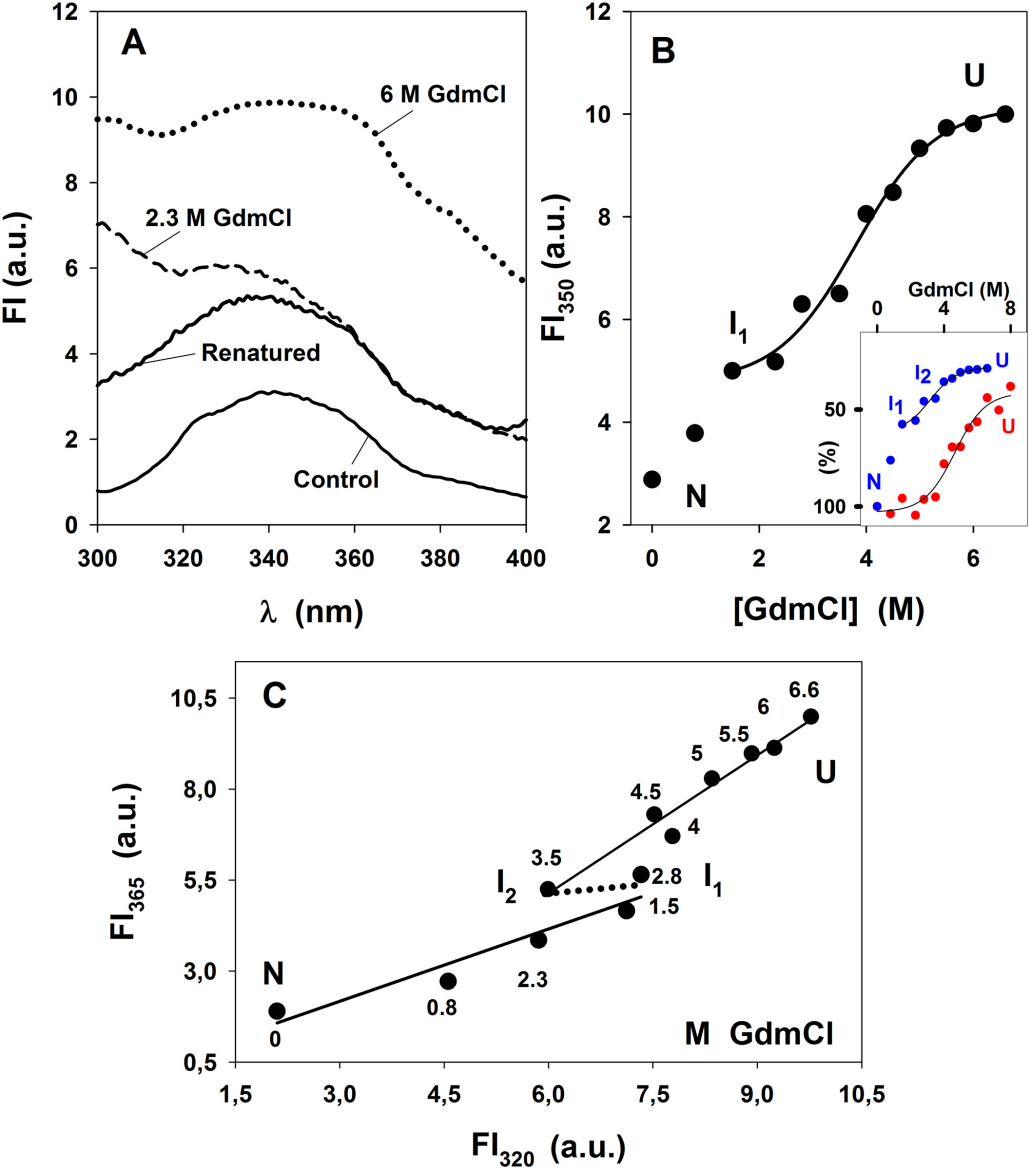
Effect of GdmCl on the fluorescence emission of SP-B_N_. (A) Fluorescence intensity emission (FI) of the propeptide in 5 mM AMT buffer, 150 mM NaCl pH 7 with 0–6.6 M GdmCl after excitation at 290 nm *vs* the wavelength. Spectrum without GdmCl (control, solid line), with 2.3 M GdmCl (dashed line), 6 M GdmCl (dotted line) or 6 M GdmCl and ulterior dialysis towards the same buffer (renatured, solid line) were recorded at 25°C. Arbitrary units (a.u.) were employed. (B) Variation of the fluorescence emission at 350 nm with 0–6.6 M GdmCl. The line is a fit of data in the 1.5–6.6 M interval to Eq (1) (r = 0.99). Inset: Comparison of secondary structure (% [θ]^220^, red circle, data and fitting from Fig 1B) and tertiary structure (FI_350_N·(FI_350_)^-1^·10^2^), blue symbol, data and fitting from Fig 3B, N is the native form). (C) Phase diagram of 0–6.6 M GdmCl based on the fluorescence emission of the propeptide at two wavelengths. Solid lines are the fit of experimental data by linear regression: 0–2.8 M (r = 0.96) and 3.5 to 6.6 M (r = 0.98). Dotted line (2.8–3.5 M) is not a fit. I_1_ and I_2_ are intermediate states in the protein GdmCl-induced unfolding; N and U as in Fig 1. Numbers beside the points are denaturant concentration.

D_1/2_ is the denaturant concentration at the midpoint transition in the [θ]^220^ (deg·cm^2^·dmol^-1^) or FI_350_ (nm) dependence on denaturant. D_1/2_ values were obtained by fitting the data of Figs 1B, 2B and 3B to Eq (3). ΔG^0^_H2O_ and *m* values were obtained by fitting the same data to Eq (2). N, U and I mean native, unfolded and intermediate state forms of the propeptide respectively and their appearance in parentheses indicate the protein forms holding the actual transition. I_1_ mean intermediate state of the protein when more than one is observed. ND is not determined.

The midpoint transition, D_1/2_, calculated as ΔG^0^_H2O_/*m* was 4.7 M, but the calculated standard error was higher than 70%, according to the error theory. Thus, D_1/2_ was determined by fitting the same set of data to Eq (2), an equation describing the same sigmoid but containing D_1/2_ as parameter instead of *m* and ΔG^0^_H2O_ (see Materials and Methods). The midpoint transition was D_1/2_ = 4.7±0.2 M. To detect hidden intermediate states in the GdmCl unfolding process, if any, we applied the phase diagram method, which deals with the parametric dependence of independent parameters under different experimental conditions for a protein undergoing structural transformations [25]. Fig 1C depicts the molar ellipticity of SP-B_N_ at 232 nm ([θ]^232^) *vs* the obtained at 222 nm ([θ]^222^) for 0–8 M GdmCl. All data in plot were fitted by lineal regression confirming that, regarding secondary structure, only the states N and U are detected along the propeptide unfolding process. However, some caution must be considered since the election of some parameters instead of others to create a phase diagram, may allow either to detect or to miss intermediate states as has been reported for other proteins [25].

The secondary structure of SP-B_N_ was also studied in the presence of 0–7.5 M urea by far-UV CD spectroscopy (Fig 2).

The CD spectra of SP-B_N_ are plotted in Fig 2A for control sample (solid line), with 2 M urea (dashed line) and with 7.5 M urea (dotted line). The spectrum at 2 M urea exhibits more negative [θ]^220^ than control with the concomitant increase of the α-helix content, which suggests the existence of a stabilized intermediate state in the protein unfolding process regarding secondary structure. The CD spectrum recorded after dialysis of the denaturant at 4°C of the sample containing 7.5 M urea (renatured sample, solid line) is also shown in Fig 2A. Since the unfolding was practically reversible, the thermodynamic analysis of the CD data was possible. The dependence of the [θ]^220^ on urea concentration is depicted in Fig 2B. We see that the ellipticity becomes more negative from control to ~ 1.5–2 M urea whereas above ~ 3.5 M urea, the [θ]^220^ increases cooperatively up to 7.5 M urea. As moderate urea concentrations stabilize the propeptide, it excludes the existence of a two-state transition process regarding its secondary structure and suggests that the unfolding is reached through an intermediate state. When more than two states exist in protein unfolding, the treatment of the data is usually carried out considering a sequence of independent separate two-state transitions [26]. Therefore, only a part of the experimental data (1.5–7.5 M urea) in Fig 2B were fitted to Eq (2), being substituted [θ]^220^ in the N state (Y_N_) by [θ]^220^ in the intermediate state (Y_I_). The midpoint transition was 5.2±0.3 M urea. These results point to the existence of, at least, an intermediate state (I) at ~ 2 M urea as the propeptide goes from its native form (N) to its assumed unfolded state (U, as a clear plateau is not observed) with the increase in urea concentration. The experimental data in the 1.5–7.5 M urea range were also fitted to Eq (1) obtaining ΔG^0^_H2O_ = 12.8±3.1 kJ·mol^-1^ and *m* = 2.5±0.7 kJ·mol^-1^·M^-1^ (Table 1). The phase diagram in Fig 2C confirms the existence of the I state regarding the secondary structure since two sets of data were fitted by linear regression: 0–1.5 M urea (transition N-I) and 1.5–7.5 M urea (transition I-U)

### Effect of Chaotropes on the Tertiary Structure of SP-B_N_

The effect of chaotropes in the tertiary structure of SP-B_N_ was analyzed studying their effect on the trp environment by fluorescence spectroscopy. Although further analysis of the trp fluorescence emission change would require time-resolved measurements, we will simply use it as a reporter of the environment of the residues. The effect of GdmCl on the propeptide fluorescence intensity emission (FI) is shown in Fig 3.

The spectra of the propeptide without GdmCl (control, solid line), with 2.3 M GdmCl (dashed line) and with 6 M GdmCl (dotted line) are depicted in Fig 3A. As the protein becomes unfolded, the emission increases since the chemical unfolding modifies the environment of trp residues, which become less quenched than in the folded protein. In parallel, the maximum wavelength of emission is shifted from 338 nm in the absence of GdmCl to 345 nm with 6.6 M GdmCl (not shown), indicating a transition of the trp residues, from a hydrophobic environment inner in the protein to that of the polar aqueous medium. The spectrum of the dialyzed 6.6 M GdmCl sample (renatured, solid line) shows the shape of the control spectrum but twice its emission indicating that the reversion was only partial and approached the emission of the protein in the presence of 1 M denaturant (not shown). The effect of GdmCl on the fluorescence intensity emission at 350 nm (FI_350_) was studied since the FI is an extensive parameter, its value being proportional to the amount of the analyzed matter in the system [25] and is depicted in Fig 3B. The fit of the data obtained with 1.5–6.6 M GdmCl (Fig 3B) to Eq (2) gave a D_1/2_ = 3.8±0.2 M GdmCl which is lower than the obtained for the secondary structure with the same chaotrope (see Table 1) indicating that the loss of tertiary structure precedes the loss of the secondary one during GdmCl-induced propeptide unfolding. The comparison of the effect of GdmCl in the secondary and tertiary structure of the propeptide (Fig 3, inset) and the fact that both curves do not superimpose corroborates the existence of intermediates. The intermediates I_1_ (detected in Fig 3B) and I_2_ (detected in Fig 3B and 3C) with decreased tertiary structure, still have a significant proportion of native secondary structure. The thermodynamic parameters were obtained above ~ 1 M GdmCl since the effect of GdmCl in the propeptide was only partially reverted. The fitting of the same data in Fig 3B to Eq (1) gave ΔG^0^_H2O_ = 12.8±3.3 kJ·mol^-1^, coincident with the value obtained with GdmCl in CD experiments whereas *m* = 3.4±0.8 kJ·mol^-1^·M^-1^ is greater than estimated from the CD study, indicating that changes affecting the exposure of trp residues to the solvent show more cooperativity than those affecting the backbone of the protein. Those thermodynamic values would correspond to the transition I_1_-U, being I_1_ the intermediate form of the protein seen around 2 M GdmCl.

As the phase diagram method is a powerful tool to uncover hidden intermediates [25], the variation of FI_365_ with FI_320_ for each GdmCl concentration was determined and depicted in Fig 3C, to detect intermediate states of the protein. Two sets of data were fitted by linear regression, corresponding to GdmCl concentration ranges of 0–2.8 M and 3.5–6.6 M indicating that, in addition to the N and U forms, there are two intermediate forms (I_1_ and I_2_) of the propeptide regarding its tertiary structure. Therefore, the phase diagram detects an intermediate state (I_1_) at ~ 2 M GdmCl (also seen in Fig 3B), and another intermediate state (I_2_) at ~ 3.5 M GdmCl not seen previously. Both intermediate states, I_1_ and I_2_, hold altered tertiary structure while maintaining the secondary structure of the native state (see Fig 1B), a characteristic of the molten globule state [2]. Therefore, considering the results relative to the tertiary structure, the propeptide in the presence of GdmCl, would unfold according to a four-state transition as follows: N→ I_1_ ↔ I_2_ ↔ U, being irreversible the step N→ I_1_ under the experimental conditions we used to eliminate the GdmCl in the medium.

**Fig 4:**
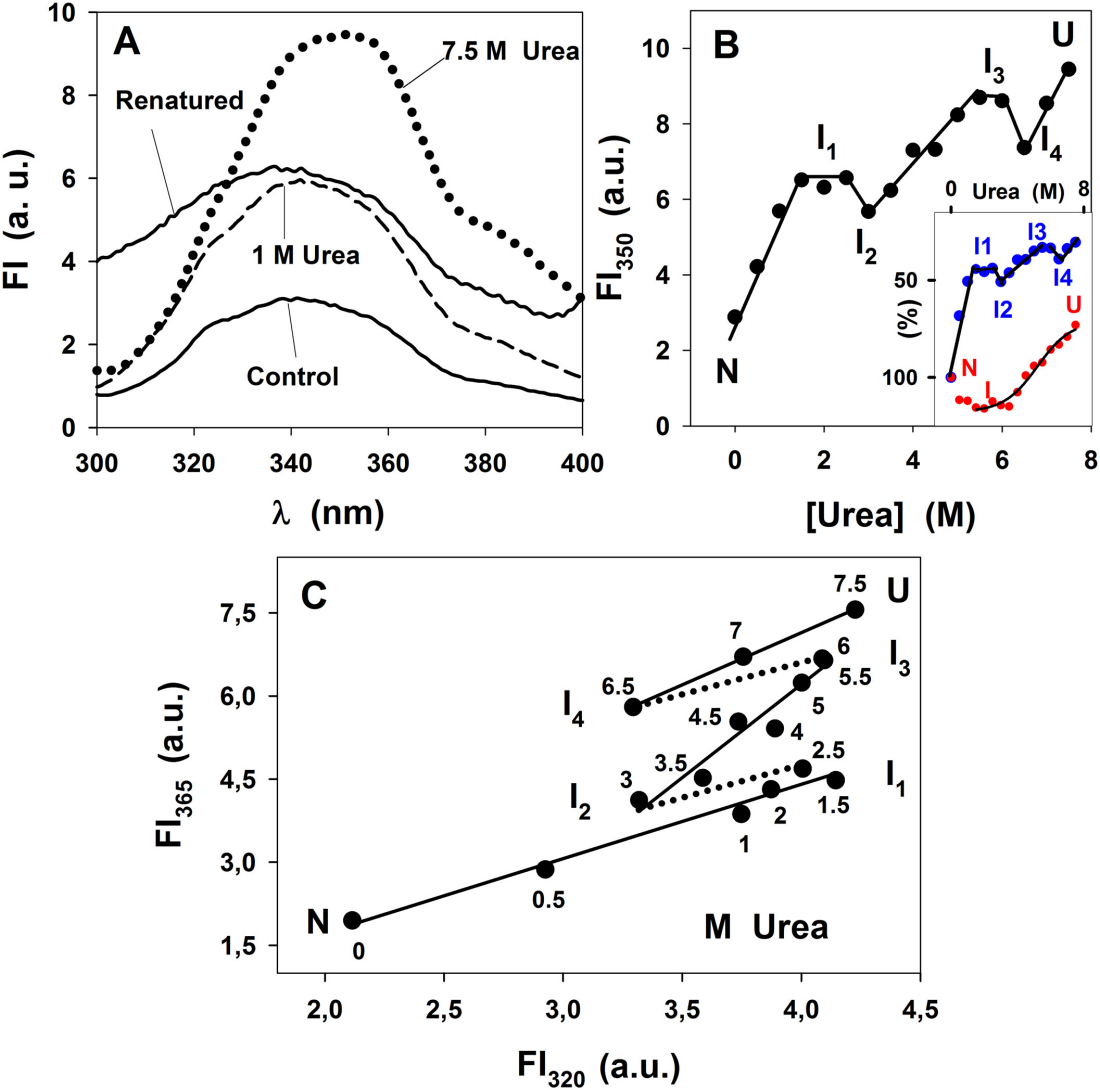
Effect of urea on the fluorescence emission of SP-B_N_. (A) Emission spectra of the protein in 0–7.5 M urea were recorded as in Fig 3. Spectra depicted are: without urea (control, solid line), with 1 M urea (dashed line), with 7.5 M urea (dotted line) and with 7.5 M urea after dialysis (renatured, solid line). (B) Variation of the fluorescence emission at 350 nm with 0–7.5 M urea. Connecting lines are not fitted. Inset: Comparison of secondary structure (% [θ]^220^, red circle, data and fitting from Fig 2B) and tertiary structure (FI_350_N·(FI_350_)^-1^·10^2^), blue symbol, data from Fig 4B being N the native form). N and U are omitted for clarity. (C) Phase diagram analysis of urea-induced equilibrium unfolding of SP-B_N_. Solid lines are fits of the data by linear regression: 0–2.5 M urea (r = 0.987), 3–6 M (r = 0.97) and 6.5–7.5 M (r = 0.999). Dotted lines are not fitted. I_1_, I_2_, I_3_ and I_4_ are intermediate states in the propeptide urea-induced unfolding. Lines are not fitted. Numbers beside the points are denaturant concentration.

The effect of urea on the tertiary structure of the propeptide is shown in Fig 4.

As described above with GdmCl, the higher the urea concentration, the greater the fluorescence emission (Fig 4A). The effect of urea on the protein could not be reverted totally (solid line, renatured). The unfolding process of the propeptide with urea is shown in Fig 4B, where no cooperative transition was observed but discontinuities such as small plateaux and valleys, pointing to the existence of several intermediate states (I_1_, I_2_, I_3_ and I_4_). The comparison of the effect of urea in the secondary and tertiary structure of the propeptide (Fig 4B, Inset) indicates that whereas I_1_ and I_2_ (observed in fluorescence spectroscopy) shows the stabilized secondary structure corresponding to I (observed in CD spectroscopy), the intermediate I_3_ conserves 92.3% of the native CD signal whereas I_4_ exhibits a more pronounced loss of secondary structure. The phase diagram in Fig 4C confirms the existence of four intermediate states, two of them at low and moderate urea as those seen at low GdmCl concentration (I_1_ and I_2_). I_3_ was observed at 5.5–6 M urea and I_4_ at ~ 6.5 M urea. The analysis of the λ_max_ dependence on urea indicates that there are four small plateaux between the N and U state (not shown), supporting the idea that the progressive exposure of trp residues to the polar medium, is reached in four steps. The high number of conformers seen in Fig 4C suggests that changes affecting the trp environment of the propeptide with urea are rather complex and compatible with a multi-state structural process. Thus regarding the tertiary structure, the propeptide would unfold with urea according to: N→ I_1_ ↔ I_2_ ↔ I_3_ ↔ I_4_ ↔U, being irreversible the N→ I_1_ process. Two of these intermediate states (I_3_ and I_4_) display near native and a remarkable loss of secondary structure respectively (Fig 2B). In contrast, I_1_ and I_2_, show also altered secondary structure, although this structure is stabilized since the [θ]^220^ is more negative (Fig 2B).

### Bis-ANS Binding Studies

**Fig 5:**
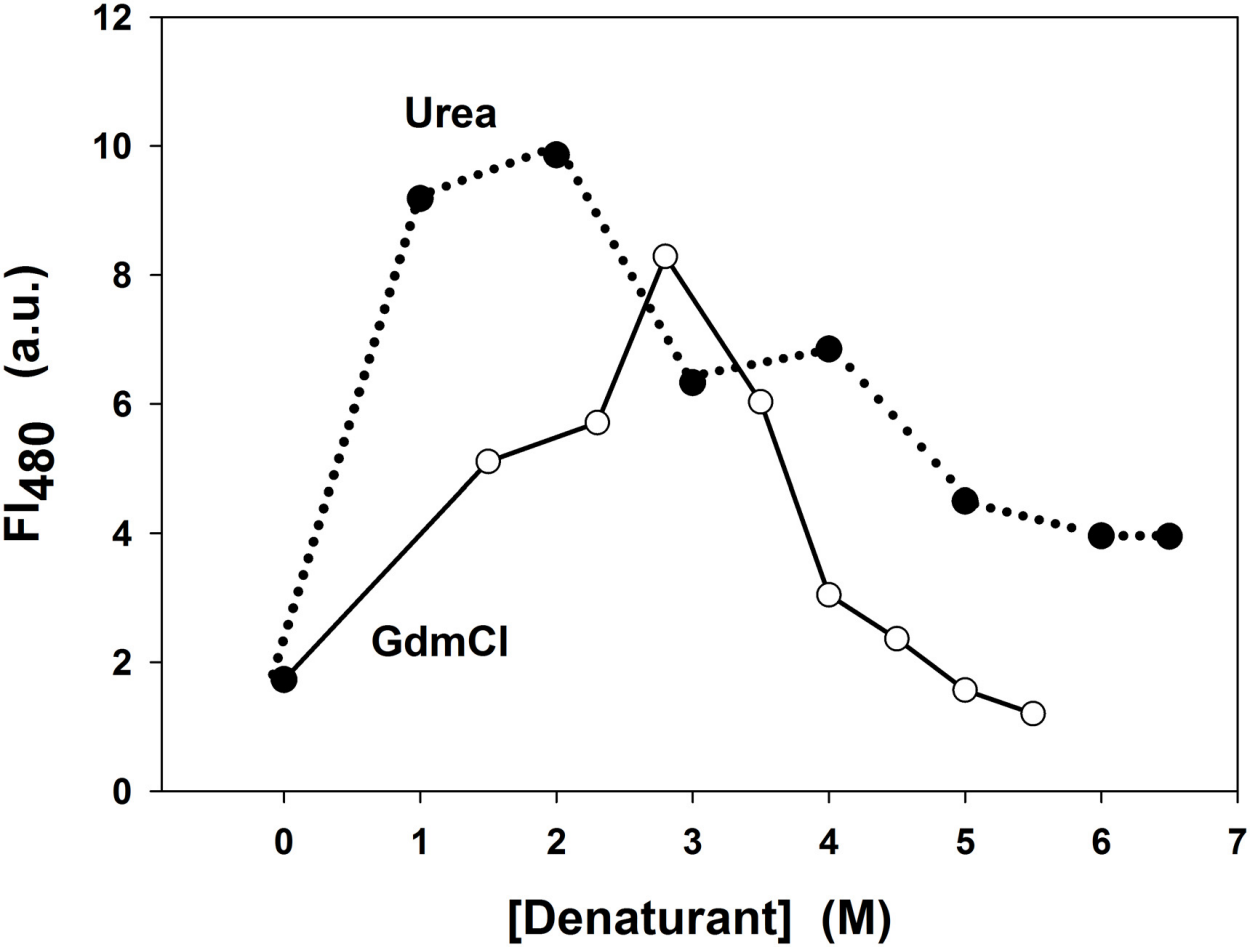
Fluorescence emission of the probe bis-ANS in the presence of SP-B_N_. Fluorescence intensity at 480 nm of the probe (11 μM) in the presence of SP-B_N_ in 5 mM AMT buffer, 150 mM NaCl pH 7 with urea (filled circles, dotted line) and GdmCl (void circles, solid line) vs denaturant concentration.

To check the molten globule nature of the intermediate states detected in the chemical-induced unfolding of the propeptide, we employed bis-ANS, a fluorescent probe that binds neither the native nor the unfolded state of proteins but is bound to intermediate structures with hydrophobic domains exposed to the medium [27]. Following the non-covalent binding of the probe to those domains, there is an increase in the fluorescence quantum yield due to the parallel orientation adopted by the two naphtyl rings of the probe. Therefore, bis-ANS can detect molten globule states of proteins holding secondary structure but with incomplete packing of the side chains in the hydrophobic core, meaning a fluctuating globular structure [28]. The effect that the environment of the propeptide has on the fluorescence emission of bis-ANS at 480 nm is shown in Fig 5.

Changes in the emission fluorescence intensity of bis-ANS were observed as urea or GdmCl concentration was increased. In the absence of denaturant, the fluorescence emission is poor but it increases with the denaturant concentration to reach maximum values at ~ 1.5–3 M (I_1_ and I_2_ intermediate states) to decrease thereafter. The decrease follows a sharp way with GdmCl as no additional intermediate state exists or in a soft way with urea according to the existence of I_3_ and I_4_ states approaching the unfolded form of the propeptide. Thus the probe bis-ANS shows emission at denaturant concentrations where intermediate states were detected, confirming them as molten globule or molten globule-like structures. Since the bis-ANS emission is higher with urea than with GdmCl, it follows that there is a higher number of exposed hydrophobic residues (where the probe can bind) in the propeptide upon unfolding with urea than upon unfolding with GdmCl.

### Effect of Temperature on SP-B_N_ Secondary Structure

**Fig 6:**
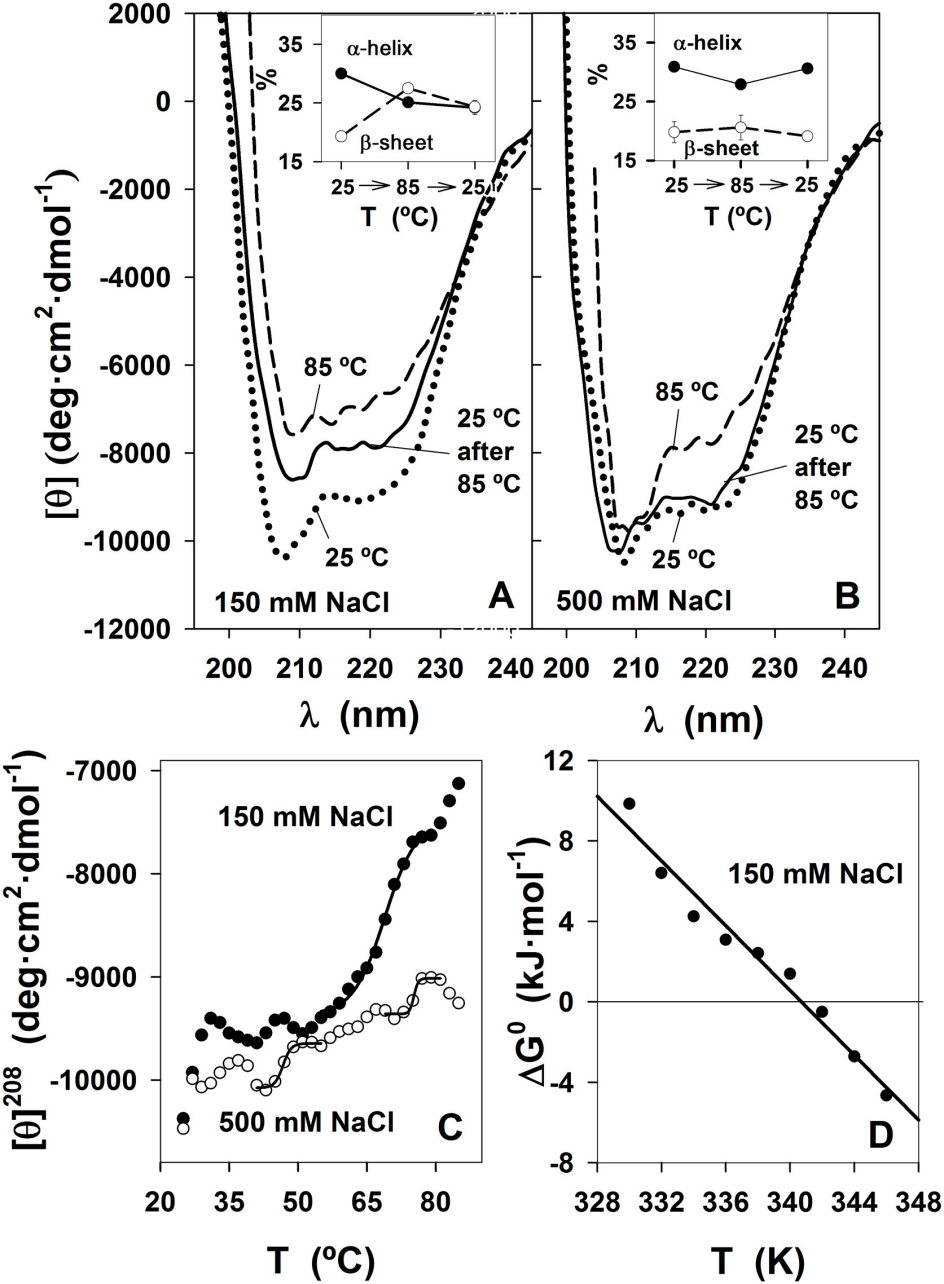
Dependence of the secondary structure of SP-B_N_ on temperature. (A) far UV-CD spectra of 0.115 mg·mL^-1^ in AMT buffer, 150 mM NaCl pH 7 at 25°C (dotted line), 85°C (dashed line) and 85°C cooled back to 25°C (solid line). Inset: Plot of α-helix (filled circles, solid line) and β-sheet (void circles, dashed line) content *vs* the temperature progress of the sample from 25°C to 85°C and back to 25°C. (B) Same as in A except that 500 mM NaCl was used. (C) Plot of [θ]^208^ along the T ramp (25–85°C; data plotted every 2°C at 150 mM NaCl (filled circles) and 500 mM NaCl (void circles). Data from 49–79°C at 150 mM NaCl were fitted to Eq (2) (solid line, r = 0.996). Data from 41–55°C (r = 0.995), 51–69°C (r = 0.981) and 69–81°C (r = 0.989) at 500 mM NaCl were also fitted to Eq (2) (solid lines through the data). (D) Dependence of ΔG^0^ on temperature according to Eq (4). ΔG^0^ values were obtained according to Eq (3) from data (49–73°C) at 150 mM NaCl except 51°C since the term (Y_N_-Y_obs_)/(Y_obs_-Y_N_) in Eq (3) was negative and the natural logarithm could not be determined. This term was also negative above 73°C. Data in plot were fitted by linear regression (solid line; r = 0.988).

The effect of temperature on the structure of the propeptide was followed by far-UV CD in the presence of 150 mM NaCl (Fig 6A) or 500 mM NaCl (Fig 6B), to check also the influence of the ionic strength on protein thermal unfolding.

The spectrum of the sample was recorded at 25°C (dotted line), then at 85°C, (dashed line) and, after the T ramp the spectrum was recorded again at 25°C (solid line). The propeptide maintains ~ 71% of [θ]^208^ with 150 mM NaCl with a decrease of 5% in the α-helix content and a parallel increase in β-sheet content (Fig 6A, inset). At 500 mM NaCl, the only change observed at 85°C is a small decrease of the molar ellipticity at 220 nm (Fig 6B) without noteworthy changes in the α-helix or β-sheet contents (Fig 6B, inset). The unfolding, whatever its extension is totally reversible after re-cooling at high salt (Fig 6B) and almost totally reversible at low salt (Fig 6A). The behaviour of SP-B_N_ with temperature was monitored by recording [θ]^208^ as shown in Fig 6C. We see that [θ]^208^ at 25°C becomes less negative upon raising the temperature to be more or less stabilized at ~ 38°C at low salt and at ~ 30°C at moderate salt, to wave again several times without reaching a clear plateau, indicative that the unfolded state was not reached at 85°C. Moreover, above ~70°C, [θ]^208^ does not increase at 500 mM NaCl as much as it does with low salt, indicating that ionic strength stabilizes the secondary structure of the propeptide. We have considered multi-state transitions as if they were consecutive independent two-state transitions as we did when the denaturant was a chaotrope, and is usually done [26]. The fitting of the experimental data to Eq (2) at 150 mM NaCl in the 49–79°C range gave a midpoint transition of 68.7±0.6°C. When 500 mM NaCl was employed, three small transitions at 41–55°C, 51–69°C and 69–81°C could be detected and fitted to Eq (2), with midpoint transitions of 46.7±0.2°C, 62.7±1.6°C and 75.2±0.4°C respectively. The observed changes in the propeptide secondary structure may be due to partial unfolding of protein domains or subdomains (small increases in [θ]^208^) or to the dissociation of propeptide oligomers [15]. Alternatively, small decreases in [θ]^208^ may be due to partial folding of unstructured domains existing in the connector arm of the propeptide (predictions in S2 Fig) as observed by Uversky in other proteins [29]. As the unfolding of the propeptide was not reached up to 87°C, its melting temperature could not be determined. The effect of temperature on the propeptide up to 85°C is practically reversible as seen in Fig 6A, thus the thermodynamic parameters for the transition at low salt could be calculated. ΔG^0^ values were obtained by fitting data of Fig 6C in the 49–79°C interval to Eq (3) at each temperature with the exception of 51°C and temperatures above 73°C as the term (Y_N_-Y_obs_)/(Y_obs_-Y_U_) in Eq (3) was negative and the natural logarithm could not be calculated. ΔG^0^ values were plotted *vs* the temperature in Fig 6D and data were fitted to Eq (4) (solid line). The intercept was the change in enthalpy for the transition, ΔH^0^ = 234.5 kJ·mol^-1^, whereas the absolute value of the slope was the change in entropy ΔS^0^ = 0.69 kJ·mol^-1^·K^-1^. Those values indicate that the predominant forces involved in the interactions of the protein domains that suffer the thermal transition or in oligomer formation, are of enthalpic character, i.e. electrostatic interactions and/or hydrogen bonding. The calculated value of ΔG^0^ at 25°C was 28.9 kJ·mol^-1^. The thermodynamic parameters of the protein in 500 mM NaCl could not be determined because of shortage of data to employ Eq (3). This was a consequence of the existence of small thermal transitions at moderate salt instead of the sole transition seen at low salt (Fig 6C).

### Hydrodynamic Properties of SP-B_N_

**Fig 7:**
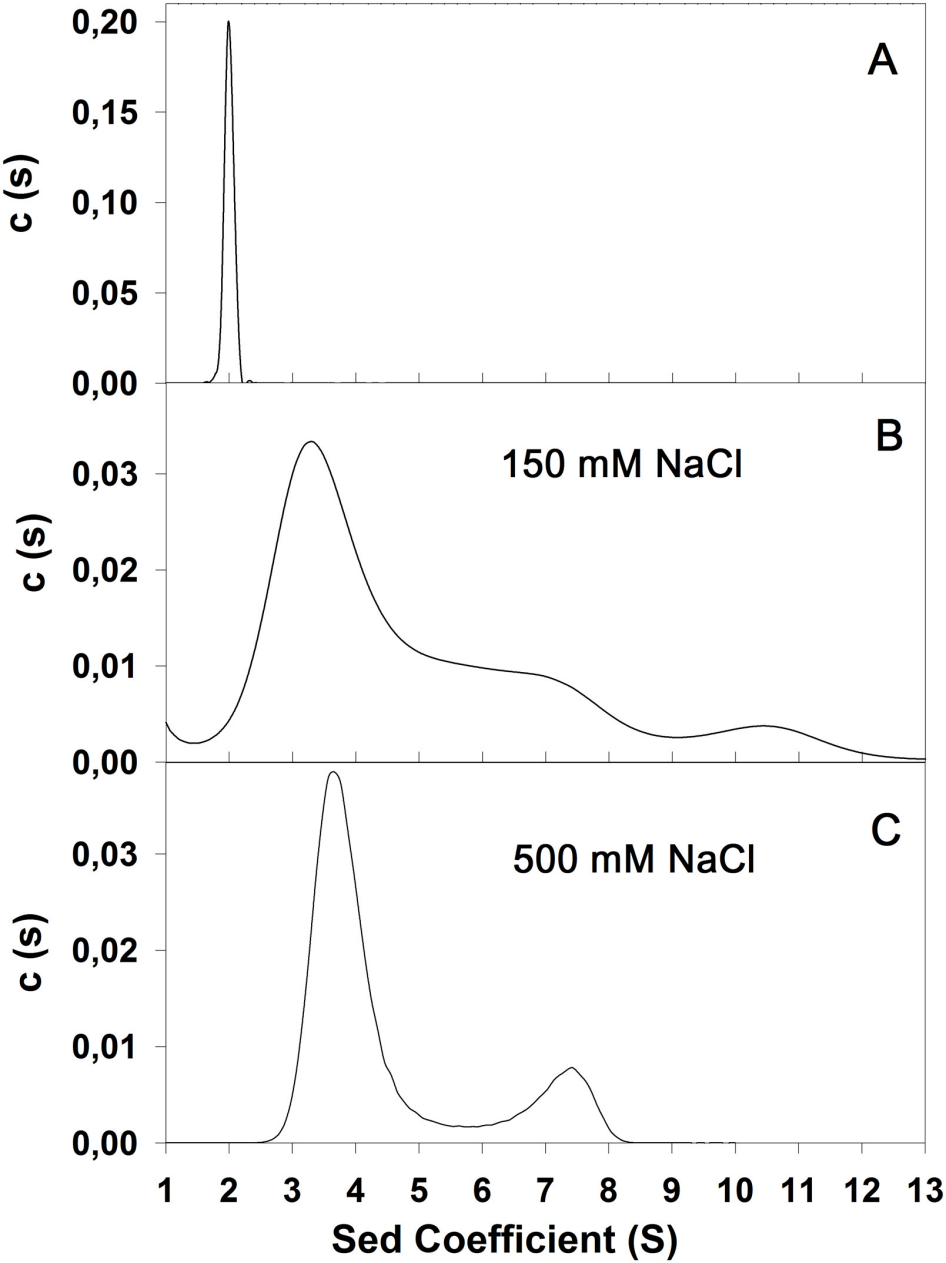
Dependence of the sedimentation velocity of SP-B_N_ on ionic strength. (A) Sedimentation coefficient distribution at 20°C and 48,000 rpm of the propeptide (0.15 mg·mL^-1^) in 5 mM Tris buffer pH 7. (B) Same conditions as described in A with 150 mM NaCl. (C) Same conditions as described in A with 500 mM NaCl.

In order to check the effect of the salt in the quaternary structure of the recombinant protein we performed boundary sedimentation experiments with 0.15 mg·mL^-1^ protein in 5 mM Tris-HCl pH 7 under different salt conditions and results are depicted in Fig 7.

The raw sedimentation coefficients (s) and the sedimentation coefficients in standard conditions, 20°C and water (s_20,w_) are summarized in Table 2.

**Table 2:** Sedimentation velocity parameters of SP-B_N_.

NaCl (mM)	Parameters			
	s (S)	s_20,w_ (S)	f/f0	appM (kDa)
	1.96±0.26	2.01±0.27	1.6	19.7
**150**	3.49±0.66	3.60±0.67	1.34	39.6
**500**	3.74±0.76	4.15±0.84	1.1	44
	6.95±0.61	7.71±0.67	1.1	123

The experimental sedimentation coefficients (s) were converted to standard conditions (s_20,w_; 20°C, water) and expressed in Svedberg. f/f0 is the frictional ratio of the protein to a sphere and is an average of the different species in the sample, and appM is the apparent molecular mass of the species.

The sedimentation coefficient distribution, c(s) in the absence of NaCl is depicted in Fig 7A. It corresponds to a symmetrical peak with s = 1.96±0.26 S and s_20,w_ = 2.01±0.27 S, corresponding to an apparent molecular mass of ~19.7 kDa which is well in line with a SP-B_N_ monomer since the molecular weight of SP-B_N_ determined by mass spectrometry is 19,883 Da [13] and the estimated from the amino acid composition is 19,902 Da. The frictional ratio f/f_0_ = 1.6 would indicate that the species has an elongated shape, equivalent to a prolate (a/b = 7.86) or oblate (a/b = 8.74) ellipsoid although we cannot discard that the extremely low ionic strength the buffer provides is affecting the species sedimentation. Moreover, only 6% of the loaded protein was detected which indicates a massive protein precipitation. In the presence of 500 mM NaCl (Fig 7C) the protein (35% of the loaded protein) sediments mainly with an apparent sedimentation coefficient of 3.74±0.76 S and 6.95±0.61 S. These peaks are compatible with dimeric (~ 40 kDa) and hexameric forms (~ 123 kDa) of the propeptide, respectively, although oligomers with another stoichiometry can coexist at lower concentration, since the peaks are not really slender. The lower averaged frictional ratio, f/f_0_ = 1.1 at this salt concentration, point to monomers associating in a side-by-side rather than end-to-end manner to form oligomers more compact than the monomer. Analysis of the sedimentation profile of SP-B_N_ was also carried out with 150 mM NaCl and results in Fig 7B indicate a great heterogeneity in the sample. The average sedimentation coefficient was only determined for the species constituting the major part of the preparation (s = 3.49±0.66 S; averaged f/f0 = 1.34; appM ~ 39.6 kDa) indicating that the dimer co-exists with several other species whose sedimentation coefficient could not be evaluated due to lack of resolution of the peaks. Moreover, the maxima in the sedimentation coefficient distribution do not necessarily corresponds to the precise values of the actual sedimentation coefficients of each species because the positions of these maxima are sensitive to the rate of monomer-oligomer association/dissociation as has been observed for other proteins [30]. Considering the sedimentation coefficients obtained in the experiment, it appears that monomers, anions at pH 7, would counteract the electrostatic repulsion between them, hiding their hydrophobic regions through mutual interaction to form dimers and higher oligomers of a globular shape, in the presence of low or moderate salt in the medium. SP-B_N_ monomers are only detected in absence of added salt although the vast majority of the protein suffers precipitation propitiated by the low ionic strength.

### Calorimetric Behaviour of SP-B_N_

**Fig 8:**
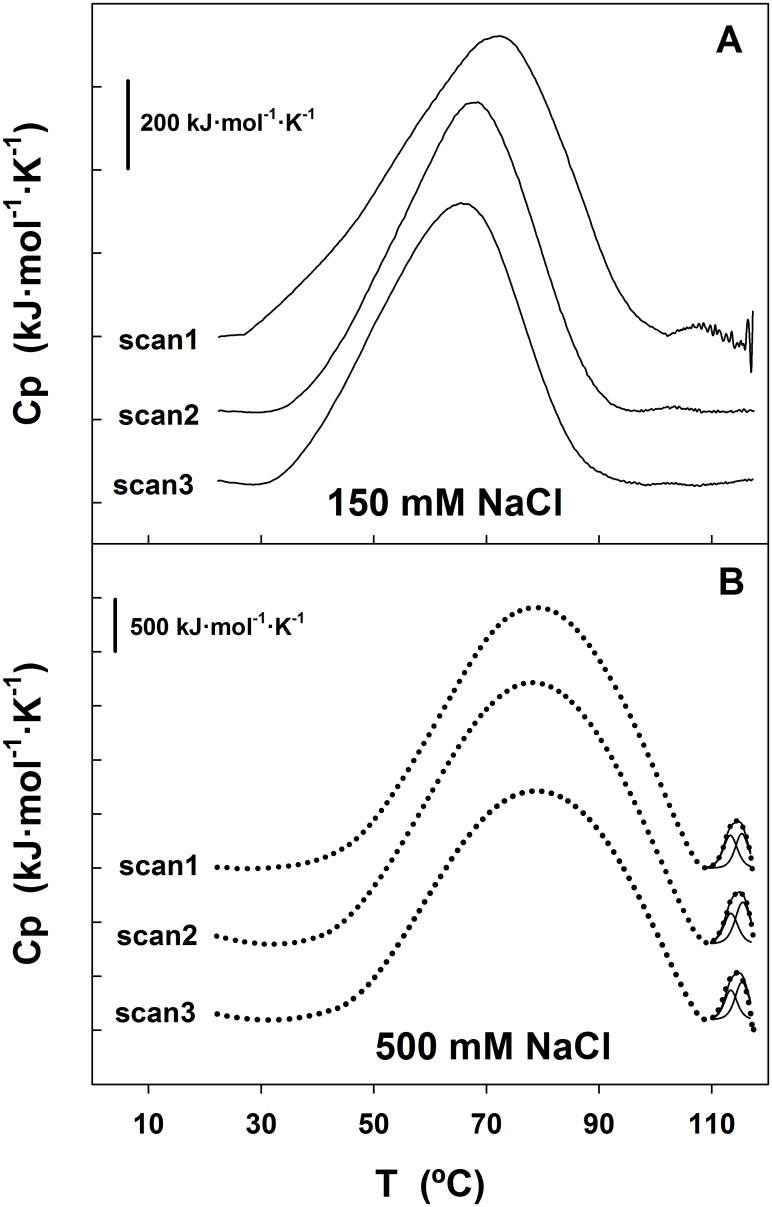
Thermal unfolding of SP-B_N_ followed by DSC. (A) Variation of the excess heat capacity (Cp) of SP-B_N_ (0.3 mg·mL^-1^) with temperature at 60°C·h^-1^ in 5 mM Tris-ClH 150 mM NaCl pH 7 (scan 1, solid line). The heated sample was cooled back, Cp recorded (scan 2), cooled again and Cp recorded (scan 3). (B) Same as in A except that the sample contained 500 mM NaCl. The dotted lines correspond to the experimental data, the solid lines through these points are the cumulative Gauss curves according to the model of Non Two-States and the other two solid lines were obtained by deconvolution analysis with Eq (5).

Differential scanning calorimetry (DSC) was employed for the thermodynamic characterization of the structural changes accompanying the unfolding process of 0.3 mg·mL^-1^ SP-B_N_ in the 20–120°C interval at 60°C·h^-1^ scan rate (Fig 8).

The variation of the excess heat capacity (Cp) of the protein with the temperature at 150 mM NaCl in Fig 8A (scan1) is characterized by two endothermic peaks whose integration gave transition temperatures that we considered as apparent T_m_ along this work: T_m1_ = 72.2°C and T_m2_ = 108.5°C respectively. After cooling and rescanning the sample twice, the first transition was observed in the successive scans with the T_m1_ moving to lower values and the calorimetric enthalpy change obtained by numerical integration of the peak (ΔH_cal_) decreasing as if less protein were available in the rescans to suffer this thermal transition (ΔH_cal_ was ~18% smaller in scan 2 and ~24% in scan 3; Table 3).

**Table 3:** Parameters of the SP-B_N_ calorimetric transitions measured by DSC.

NaCl (mM)	Transition 1		Transition 2			
	T_m1_^a^ (°C)	ΔH_cal 1_^a^ (kJ·mol^-1^)	T_m2_^a^ (°C)	ΔH_cal 2_^a^ (kJ·mol^-1^)	ΔH_VH_^b^ (kJ·mol^-1^)	E_A_^c^ (kJ·mol^-1^)
**150**						
scan1	72.2	26,269	108.5	150.4	ND	703.8
scan2	68.4	21,411	ND	ND	ND	ND
scan3	65.6	19,929	ND	ND	ND	ND
**500**						
scan1	78.8	90,899	114.4 (113.3; 115.3)^b^	1,841 (994; 981)^b^	(1,505; 1,627)^b^	ND
scan2	77.6	93,291	114.8 (113.4; 115.5)^b^	2,089 (1,352; 930)^b^	(1,422; 1,495)^b^	ND
scan3	78.7	81,413	114.5 (113.4; 115.4)^b^	1,782 (1,115; 813)^b^	(1,547; 1,649)^b^	ND

^*a*^: The apparent transition temperatures and the change in calorimetric enthalpies of the reversible and irreversible processes were determined by integrating the corresponding peak in Fig 8.

^*b*^: The van´t Hoff enthalpy, the apparent T_m_ and the calorimetric enthalpy of the subpeaks (in parenthesis) were determined by deconvolution analysis, fitting the data in Fig 8B to Eq (5).

^*c*^: The activation energy of the irreversible transition in Fig 8A was determined according to Eq (6). ND is not determined.

When two transitions are observed in the thermogram of an oligomeric polypeptide, the first one is usually attributed to oligomer dissociation and the second to monomeric protein unfolding [31]. The data corresponding to the first transition could not be fit to the oligomer dissociation equations provided by Origin. The cause may be that those Eqs apply to the dissociation of oligomers with definite stoichiometry such as dimer, hexamer, u.s.w. whereas SP-B_N_ is a complex mixture of oligomers of different sizes as seen in analytical ultracentrifugation (Fig 7C) and the endothermic peak must be due to the dissociation of more than one oligomer type. Regarding the second transition, the small peak observed in scan 1 was unobserved after rescanning, indicating that this calorimetric process is irreversible. There is a small exothermic contribution following this endothermic peak, which suggests that the protein is suffering unfolding. Moreover, the noisy traces seen at higher temperatures are likely due to convection of clump aggregates into the DSC cell. Hence, it is probable that aggregation of unfolded protein causes the irreversibility. As there is no clear exothermic peak, we suggest that the cooling following unfolding might allow a partial refolding which is seen, after rescanning, only in the first transition as oligomers of distinct (and greater) n-mers dissociation. We tried to find if the aggregation process was under kinetic control by varying the scan rate but the thermograms obtained at 15°C·h^-1^ and 30°C·h^-1^ showed also protein aggregation at higher temperatures (not shown). Therefore, the dependence of T_m2_ on the scan rate could not be established. The activation energy for the irreversible transition (one-step process from native to irreversibly inactivated state of the protein) was obtained with Eq (6), E_A_ = 703.8 kJ·mol^-1^ which is a high value and points to the protein showing a high barrier for the inactivation.

To study the influence of the increased ionic strength in the calorimetric behaviour of SP-B_N_, the experiment was repeated under the same conditions except that 500 mM NaCl were employed. Results are depicted in Fig 8B. The endothermic peaks observed in the thermogram in scan 1 were also observed through rescanning the sample, their T_m_ being practically the same (Table 3) which indicates that both thermal transitions are reversible. Comparing the T_m_ values obtained in scan 1 at 500 mM NaCl (78.8°C and 114.4°C) with the obtained at 150 mM NaCl (72.2°C and 108.5°C), we see that the increase in ionic strength shifts both T_m_ to higher temperatures. It appears that the protein has become stabilized by the salt and this effect is also reflected in ΔH_cal_ values which are higher at 500 mM NaCl than at 150 mM NaCl (Table 3).

The reversibility of the second calorimetric transition allowed the thermodynamic treatment of the data. As the Cp curve reflects the energetic variations associated with differently populated states in thermodynamic equilibrium with each other, the number of intermediate states and the thermodynamic variables associated with the second peak were calculated by using an appropriate mechanical-statistical deconvolution algorithm [32]. The reversible transitions at each successive scan were subjected to thermodynamic deconvolution analysis and the experimental data were fitted to the Non Two State model with two peaks which accounted for the best fitting (the lowest χ^2^ / DoF) according to Eq (5). This indicates that SP-B_N_ does not unfold following an all-or-none transition between the native and the denatured form but that some domains in the protein are thermodynamically more stable than others and melt at specific temperatures.

The ΔH_cal_, the van´t Hoff enthalpy (standard enthalpy change of a one-step reaction; ΔH_VH_) and the apparent T_m_ for the two subpeaks obtained after the deconvolution process are summarized in Table 3. We must remark that the deconvolution algorithm employed do not take into account the eventual change in molecularity during the process. The enthalpies ratio ΔH_VH_/ΔH_cal_ is 1.51 and 1.65 for both subpeaks of scan 1 (Fig 8B and Table 3). These values, above 1, indicate that the cooperativity unit for SP-B_N_ unfolding is not the monomer but an oligomer, that is, the real stoichiometry of the protein is higher than we have assumed (the monomer molarity was used in the normalization during data processing). Therefore, intermolecular processes seem to be involved not only in the first but also in the second thermal transition. The average of the ratio values mentioned above: ΔH_VH_/ΔH_cal_ ~ 1.58, is fairly close to 1.6 which is the value calculated for the dissociation of a tetramer (n = 4) according to the equation ΔH_VH_/ΔH_cal_ = 2n (n+1)^-1^. The second transition would involve tetramer dissociation, coupled or not, to protein unfolding. However, some reservations must be made to the ratio ΔH_VH_/ΔH_cal_ as indicative of tetramer dissociation: first, we cannot discard that oligomers holding different molecularity coexist and suffer this thermal transition, and second, the unfolding reaction may also contain small irreversible and not detected steps [33].

**Fig 9:**
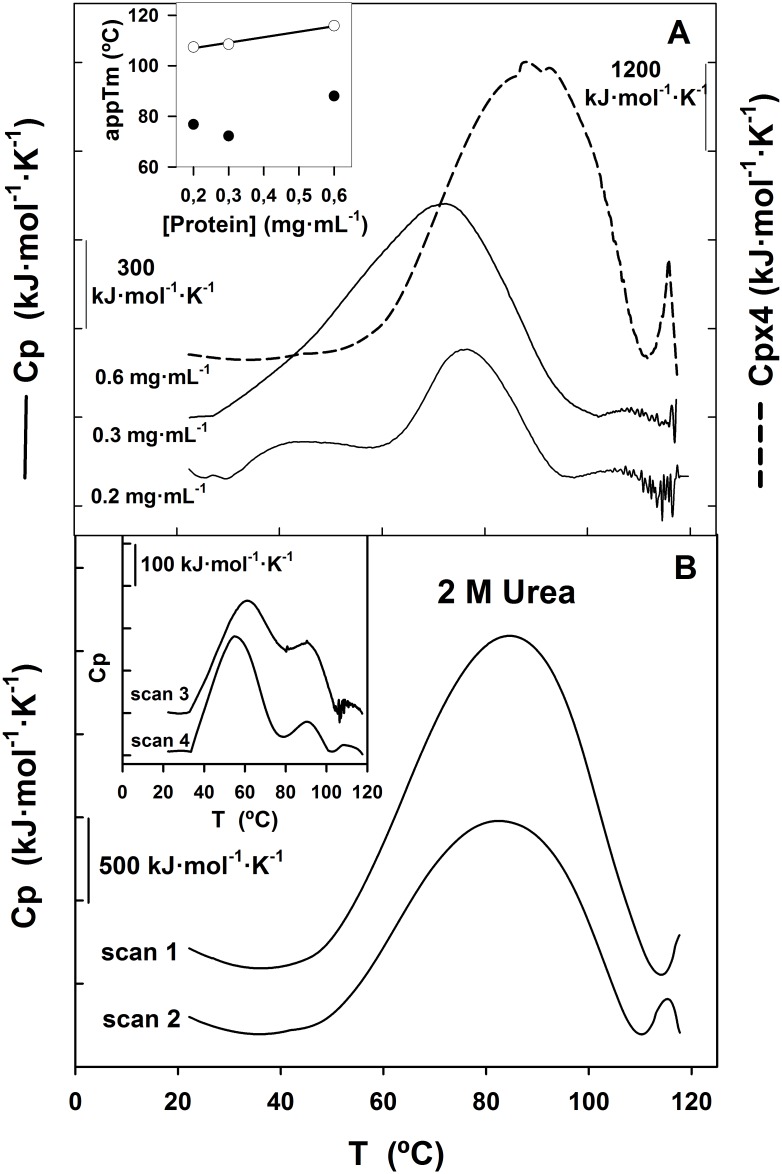
Effect of protein concentration and urea on the thermal behaviour of SP-B_N_ followed by DSC. (A) Thermograms (scan 1) of 0.2 and 0.3 mg·mL^-1^ protein (solid lines) and 0.6 mg·mL^-1^ protein (dashed line) in 5 mM Tris-ClH 150 mM NaCl pH 7 at 60°C·h^-1^. Inset: Dependence of the apparent T_m1_ (filled circles) and apparent T_m2_ (void circles) on protein concentration. The line is the fit by linear regression (r = 0.993). (B) Thermograms (scan 1 and scan 2) of 0.6 mg·mL^-1^ protein in 5 mM Tris-ClH 150 mM NaCl pH 7 with 2 M urea at 60°C·h^-1^. Inset: scan 3 and scan 4 of the same experiment.

Once established the reversible condition of the second transition at 500 mM NaCl, we searched the character of the irreversible condition of the same peak at 150 mM NaCl (see Fig 8A). To elucidate if the irreversibility was due to intermolecular interactions taking place between protein molecules, we studied the dependence of the T_m_ on protein concentration as well as the effect of 2 M urea on the T_m_ (Fig 9).

The propeptide (0.2 and 0.6 mg·mL^-1^) was subjected to DSC with any other experimental condition as described for 0.3 mg·mL^-1^ (Fig 8A) and results obtained were depicted in Fig 9A. The thermogram of 0.2 mg·mL^-1^ protein shows an endotherm (T_m1_ = 76.8°C) with a broad shoulder ~ 46°C which suggests that oligomers of several sizes are dissociating at temperatures quite apart at low protein concentration. The second peak (T_m2_ = 107.4°C) shows a noisy trace and is followed by an exothermic contribution pointing to protein aggregation. The 0.6 mg·mL^-1^ thermogram exhibits two endotherm peaks with T_m1_ = 81.7°C and T_m2_ = 115.8°C being the sharp profile of this peak quite different to the observed at lower protein concentrations. If the first thermal transition is due to oligomers dissociation and the second only to protein unfolding, the increase in protein concentration would increase only T_m1_ while it would not affect T_m2_ [34]. Regarding the first transition in Fig 9A, the T_m1_ is higher with 0.6 than with 0.3 mg·mL^-1^ protein but the different profile the endotherm shows with 0.2 mg·mL^-1^ (because of the shoulder) avoids the whole comparison. The T_m2_ increases linearly with protein concentration (Fig 9A, inset, void circles) indicating that the process includes a bimolecular step and that oligomer dissociation contributes to this thermal transition which may include or not, protein unfolding.

Seeking further confirmation, DSC experiments were carried out with 0.6 mg·mL^-1^ protein in the presence of 2 M urea and 150 mM NaCl at 60°C·h^-1^. Mild urea concentrations are usually employed to break intermolecular protein associations without affecting protein unfolding. The position of the T_m_ is strongly influenced by the molecularity (n), the T_m_ being moved towards higher temperatures as the molecularity decreases [35]. As 2 M urea has a dissociating effect on oligomers, their molecularity would be reduced and T_m1_ would increase whereas T_m2_ would be affected only if oligomer dissociation is involved in the second thermal transition. We see in Fig 9B (scan 1) that T_m1_ = 84.6°C and thus, it is increased regarding the control value at the same protein concentration (Tm1 = 81.7°C, Fig 9A, dashed line), as was expected. This value is displaced to lower temperatures in the rescans: 82.5°C (scan 2), 61.2°C (scan 3, inset), 54.7°C (scan 4, inset), which indicates that after cooling and reheating, oligomers of higher molecularity are being formed trough protein association as it has been proposed for other proteins [36]. Moreover, scan 3 and scan 4 also shows shoulders at 93°C and 91.5°C respectively indicating that oligomers of lower molecularity are being segregating from the bulk in the dissociation process. The ΔH_cal_ of the first transition also decreases regarding control (Fig 9A) with urea, in scan 1, and in the successive rescans (see the scales in Fig 9B).

Regarding T_m2_, it could not be calculated in scan 1 since urea displaces the thermal transition towards higher temperatures and the peak is no longer seen. After rescanning the sample, the peak is again in the temperature range of the calorimeter, being T_m2_: 115.3°C, 110.5°C and 108.7°C in scan 2, scan 3 and scan 4 respectively. It appears that mild urea has disrupted whatever the oligomer/s contributing to this thermal transition in its absence. Upon successive rescans, the protein would associate forming the oligomers responsible of the second thermal transition, the T_m2_ being displaced towards the low temperature side of the thermogram. From the experiments with 2 M urea, it follows that the real T_m_ of the monomeric propeptide may be higher than 117°C and is not detected in the temperature limits of the calorimeter. Attempts of determining the calorimetric behaviour of the monomeric propeptide (0.3 mg·mL^-1^, 60°C·h^-1^) in the absence of salt, were useless since the protein is prone to aggregate at those conditions.

### Free Thiol Titration

The determination of free thiols in SP-B_N_ with DTNB, with or without 5.7 M GdmCl, gave less than and 0.15 ± 0 mol cys per mol of protein indicating that the five possible disulfide bridges have been formed in the propeptide. The titration of the fusion protein, MBP-SP-B_N_ gave 10.22 ± 0.4 and 1.96 ± 0.04 in the presence and absence of 5.7 M GdmCl respectively, indicating that no disulfide bond has been formed in the fusion protein although there are two cys accessible to the reagent and eight hidden to it, in the absence of the denaturant. The protein MBP released from the fusion by proteolysis with FXa contains no cys and its titration yielded in fact 0.53 ± 0.08 mol cys per mol of protein. Therefore, the ten cys determined in the fusion belong to the propeptide indicating that whereas SP-B_N_ remains attached to MBP, no disulfide bond was formed. MBP possesses a natural protein-binding site, which is employed naturally to interact with proteins involved in maltose signalling and whose relevance to bind the fused polypeptides has been already proposed [37]. It is possible that SP-B_N_, as a partner of MBP under the reduced conditions during purification, become sequestered in a way that prevents the formation of disulfide bridges once the reducing agent was eliminated by dialysis. After cleavage of the fusion by Factor Xa, O/N at room temperature, the propeptide would fold forming 5 disulfide bonds. MBP has been proposed to act as a molecular chaperone in the context of fusion proteins by binding reversibly to aggregation-prone folding intermediates of passenger proteins to prevent their self-association [37]. MBP could then efficiently protect SP-B_N_ of undesirable folding and aggregation until, once released from the fusion by proteolysis, the propeptide would be able to fold both independent and properly.

## Discussion

The folding of a protein comes from a balance of forces between the interaction of the protein with itself and the interaction with the medium. The disruption of this balance by denaturing environmental conditions (heat, acid, alkali or organic denaturants among others) is followed by protein unfolding [38]. The folding of small proteins occurs mainly in a highly cooperative fashion (two-state process) without the presence of detectable populated intermediates [39]. In contrast, larger proteins with more than 100 residues in size usually fold in three-state transitions involving populated intermediates containing structured regions corresponding to domains or subdomains in the native state proteins [40]. At equilibrium conditions, a protein molecule can be transformed in one of several compact forms, the highly ordered molten globule (ho-MG, also named N*), the molten globule (MG), the swollen MG (MGsw) and the pre-molten globule (pre-MG, one structural form of the coil state) [3, 25]. They are different structural conformers of the protein but the MG is being considered as the only phase state existing in addition to the native (N) and the unfolded (U) states [4]. The size of the recombinant NH_2_-terminal propeptide of the precursor of SP-B, holding 177 amino acid residues, can sustain folding through a non two-state process. Statistical analysis of 154 proteins carried out by Uversky conclude that the set of intermediate(s) forming proteins shows a mean hydrophobicity of 0.446 ± 0.023 whereas 0.422 ± 0.017 was obtained for the set of proteins unable to adopt a partially folded conformation [41]. The mean hydrophobicity of the propeptide, is 0.456 which is compatible with the propeptide having a theoretical tendency to form intermediate(s) states. Moreover, when both the mean hydrophobicity and the mean net charge (0.0708) of the propeptide are combined, the protein would be located in the area occupied by the proteins folding with intermediate states within the Uversky charge-hydrophobicity phase space [41].

To explore this possibility and to detect the possible existence of intermediate states along the process, urea and GdmCl have been used to follow the unfolding transitions of SP-B_N_ at 25°C through changes in secondary and tertiary structures. Employing the phase diagram method, intermediate states at ~ 2 M and 3.5 M GdmCl have been detected showing native-like secondary and altered tertiary structures. They could correspond to the ho-MG (N*) and MG states respectively as they bind to bis-ANS. Four intermediate states were detected at ~ 2 M, ~ 3 M, ~ 5.5 M and 6.5 M urea, all of them holding more or less altered tertiary structure and binding bis-ANS, which confirms their character of MG-like states. At low and moderate urea, the scenario is more complex since an increase in negative ellipticity points to protein stabilization (intermediates I_1_ and I_2_) as other proteins have shown [42]. At higher urea, I_3_ shows native-like secondary structure and is compatible with the MG state whereas I_4_ shows ~ 80% of the CD signal approaching the pre-MG state, which would be half-way between the MG and the U states and is characterized by holding destabilized secondary structure (~ 50% of the N structure) and weaker interactions with bis-ANS [4].

Unfolding is a thermodynamic process involving a change in free energy and changes in conformation between the native and the unfolded states [43]. To quantify protein stability, the conformational stability is commonly used (the Gibbs energy change; ΔG^0^_H2O_), which is used to compare stabilities of closely related proteins [44]. Relative to the actual validity of ΔG^0^_H2O_ values obtained from unfolding transitions with chaotropes, it happens that, for an increasing number of proteins, the free energy change derived from hydrogen exchange experiments has been shown to be equivalent to the values obtained from calorimetric data or using spectroscopic probes [45]. The ΔG^0^_H2O_ of the propeptide is ~ 12.7 kJ·mol^-1^ in CD assays (with any chaotrope) or in fluorescence emission assays (with GdmCl), suggesting that chemical induced unfolding deals with a same basic phenomenon and that the distribution of species, which comprises the unfolded state, must be the same in both modes of unfolding. This ΔG^0^_H2O_ is relatively low compared with the values reported for common globular proteins whose native state appears to be stabilized by 17.7–83.6 kJ·mol^-1^ [46] or 21–41.8 kJ·mol^-1^ [47]. Thereby, the propeptide is only marginally stable, which may be necessary to achieve its biological function, either chaperoning mature SP-B or potentially acting in the vicinity of membranes. The *m* values (a measure of the cooperativity of the denaturation) reflect the change in solvent exposure of the protein during the transition [47]. As the *m* values are close in CD assays with GdmCl and urea in the N-U and I-U transitions respectively, it follows that changes affecting the backbone of the protein show the same cooperativity disregarding the denaturant whereas those affecting the exposure of trp residues to the solvent with GdmCl are more cooperative since *m* is higher.

The effect of temperature on the propeptide secondary structure detected several thermal transitions, none of them leading to T_m_ determination since thermal unfolding was not achieved. Instead, these transitions exhibited midpoints around 68°C at 150 mM NaCl and 46°C, 62°C and 75°C at 500 mM NaCl. It is known that multi-domain proteins usually unfold step-wise, with the domains unfolding individually, either independently or with varying degrees of interactions between them whereas in oligomeric proteins, the subunits dissociation usually precedes the monomer unfolding [48]. Therefore, the thermal transitions observed in SP-B_N_ may correspond to the unfolding of certain protein domains or subdomains and/or to oligomers dissociation whereas the overall protein shows resistance to temperature-induced unfolding up to ~ 86°C. DSC studies at 150 mM NaCl evidenced two transitions, one reversible with apparent T_m1_ = 72.2°C due probably to oligomers dissociation and perhaps to subdomains unfolding and a second irreversible transition with apparent T_m2_ = 108.5°C. Results obtained with different protein concentrations or with urea, point to oligomers dissociation taking place in both transitions. The irreversible character of the process causing the second peak at 150 mM NaCl disappeared at 500 mM NaCl, suggesting that Na ions could weak the repulsive forces the propeptide holds at neutral pH (pI = 4.45) favouring compaction and oligomerization and hindering aggregation at high temperature. The fitting of data in the second transition of the reversible process to Non Two-States with two subpeaks equation confirms the existence of intermediate states in the process and the ratio ΔH_VH_/ΔH_cal_ ~ 1.6 of the subpeaks suggests the dissociation of tetramers, maybe in addition to protein unfolding. Sedimentation data obtained at this salt concentration detected mainly species compatible with dimers and hexamers but the lack of slenderness of the peaks points to other coexisting species whereas crosslinking experiments (S3 Fig) evidenced dimers and trimers.

The high activation energy for the irreversible transition (E_A_ = 703.8 kJ·mol^-1^, Fig 9A and Table 3) indicates that a big energy barrier is present in the propeptide making its unfolding slow and irreversible since the E_A_ for many proteins is in the 100–500 kJ·mol-1 range [49]. The thermal resistance of the propeptide to unfold suggests stabilization by intrahelical disulfide bonds and ulterior confirmation comes from the impossibility of titrating thiol groups in cys residues with or without 5.7 M GdmCl. Thus, the propeptide (S1 Fig) must hold three disulfide bonds in the SAPB domain, by homology with those existing in saposins, which provides them with extreme stability against thermal denaturation and degradation by proteases [16, 17]. The SAPB module of the propeptide has been isolated from rat lung and may display, by homology, a disulfide bond pattern according to the exhibited by saposins [12]. The exact localization of the remaining two bonds in the SAPA domain of the propeptide is still to be determined. The structure of the different intermediates in the folding pathway could reflect different dispositions and compactions of the SAPB module (taking ~ 1/2 of the total sequence of SP-B_N_) with respect to their flanking segments (SAPA module and the connector arm). Denaturation of SP-B_N_ induced by urea or GdmCl ends in structures still preserving a substantial amount of secondary structural elements (CD experiments) which could be concentrated in the disulfide-cross-linked SAPB module. This seems to indicate that the disulfide bonds formed according to the expected saposin pattern could provide a surplus of stability to the core (probably the most important part, from a functional point of view, of the protein). In addition, the acidification of the medium causes the formation of a coiled-coil structure with a stretch of the SAPB domain involved in it [15]. Moreover, the presence of salt in the buffer may be important for neutralizing the acidic residues (28%) concentrated in the ^148^P-D^174^ stretch of the connector arm, where the protein must hold disorder, for promoting stabilization upon oligomerization.

This complex scenario of protein unfolding is likely related with the role of the N-terminal propeptide to modulate processing and assembly of mature SP-B into pulmonary surfactant complexes, along the pathway of surfactant biogenesis in type II pneumocytes. We propose that the intermediates resolved in the structure of SP-B_N_ could reflect true intermediate structures with a functional meaning once processing of proSP-B is triggered in acidic subcellular compartments, in the presence of phospholipìd surfactant membranes. Partly folded intermediates may be required to expose disulfide-stabilized saposin domains for assembly into membranes, involving or not changes in the oligomerization state, as a consequence of conformational changes affecting the connector segments. Proteolysis targets are located within those intervening sequences, suggesting that partial unfolding, which could be originated by acidified pH under the physiological context of pneumocytes, could be strictly necessary to trigger maturation and assembly. This is relevant not only to understand the complex process of surfactant assembly in vivo, but to optimize the production of surfactant protein SP-B analogues by recombinant strategies, something that still remains elusive. Our results suggest that processing of recombinant propeptides in vitro, an apparently limiting step, may need coupling proteolytic maturation with conditions facilitating the acquisition of the partly folded molten globule-like intermediates that expose both the cleavage sites and the hydrophobic membrane-active modules.

## Supporting Information

S1 Fig Scheme of human preproSP-B structure. *Numbers* indicate the amino acid position at the beginning and end of the Saposin A-type like module (SAPA) or the B-type (SAPB). Signal peptide encompasses amino acids 1–23, NH_2_-terminal propeptide: 24–200 and mature SP-B: 201–279. Solid lines between cysteine residues indicate disulfide bonds and dashed lines indicate putative disulfide bonds by homology with bonds in saposin modules of preproSaposin. Red cys in mature SP-B serves to dimerize mature SP-B through interchain disulphide bond. Potential glycosilation site in the NH_2_-terminal propeptide (N^129^) is omitted as the protein is produced in bacteria. The second site (N^311^) in the COOH-terminal propeptide is also omitted.
(DOC)

S2 Fig Prediction of disordered regions in SP-B_N_. Disordered regions in SP-B_N_ have been predicted by Spritz v0.1 [1]. The total % disorder is 25.42, comprising three patches of 4, 22 and 19 amino acids respectively as is shown in the propeptide sequence (D means disorder and O means globular). The bigger patches correspond to connector arm. [1] Vullo A, Bortolami O, Pollastri G, Tosato SC, Spritz A: a server for the prediction of intrinsically disordered regions in protein sequences using kernel machines. Nucleic Acids Res. 2006;34: 164–168.
(DOC)

S3 Fig Time progress of SP-B_N_ cross-linked oligomers formation. The propeptide was incubated with 0.08% (v/v) glutaraldehyde (+GA) for 20 s and 120 s or without it (-GA) at 20°C (control sample, ~ 20 kDa). The positive control was 2 μg of the propeptide fused to the Maltose Binding Protein (F, ~ 62 kDa). To confirm and clarify the SP-B_N_ oligomers, we carried out experiments with the bifunctional cross-linking agent glutaraldehyde. SP-B_N_ samples (0.26 mg·mL^-1^) in 20 mM Tris-HCl, 500 mM NaCl pH 7.0 were incubated at 20°C with 0.08% (v/v) glutaraldehyde (GA, Amersham) during different times. This is a protein concentration above the concentration employed when only intramolecular crosslinking is desired (~ 0.1mg·mL^-1^) [1]. The reaction was finished by addition of 5 μL of loading buffer (5x; SDS, reducing conditions) to 20 μL of the sample and 5.2 μg of protein was applied to 12% SDS-PAGE. SP-B_N_ was detected by immunoblotting with a primary monoclonal anti-proSP-B antibody (a gift of Dr Weaver, University of Cincinnati, USA) and bound antibody was probed with a secondary anti-Mouse IgG peroxidase-conjugate antibody (Sigma) as described [2]. The recombinant SP-B_N_ in absence of GA shows a band of ~ 20 kDa corresponding to the monomer and a trace band migrating as the positive control (fusion protein) which is seen due to deliberate over-exposition. Incubation of SP-B_N_ with 0.08% (v/v) GA for 20 s and 120 s decreased progressively the ~ 20 kDa band signal whereas new band signals were detected Those bands were assigned to putative dimer and trimer cross-linked forms of SP-B_N_ respectively. In samples exposed to higher GA concentrations or incubated longer times at the same concentration, SP-B_N_ was cross-linked to produce higher molecular mass species which either did not enter or remained at the non-lineal part of the gel (not shown). Attempts of detecting any SP-B_N_ cross-linked species staining the gel with Coomassie, failed due to the small quantities of oligomers entering the gel. By the other hand, lower GA concentrations than 0.08% (v/v) were not effective to crosslink the protein. [1] Ohno H, Kurusu F. Cytochrome C cross-linked with glutaraldehyde. Electrochemical response in poly(ethylene oxide) oligomers. Chem Lett. 1996;8: 693–694. [2] Palacios A, González B, Alonso S, Pérez-Gil J, Estrada P. Production of a recombinant form of the propeptide NH_2_-Terminal of the precursor of pulmonary surfactant protein B. Enzyme Microb Technol. 2006;40: 85–92.
(DOC)
