# Supplementary material for: Conformational Stability of the NH2-Terminal Propeptide of the Precursor of Pulmonary Surfactant Protein SP-B
Source: PLoS One. 2016 Jul 5;11(7):e0158430. doi: 10.1371/journal.pone.0158430 (PMC4933373; doi:10.1371/journal.pone.0158430)
Supplement: S1 Fig — Numbers indicate the amino acid position at the beginning and end of the Saposin A-type like module (SAPA) or the B-type (SAPB). Signal peptide encompasses amino acids 1–23, NH2-terminal propeptide: 24–200 and mature SP-B: 201–279. Solid lines between cysteine residues indicate disulfide bonds and dashed lines indicate putative disulfide bonds by homology with bonds in saposin modules of preproSaposin. Red cys in mature SP-B serves to dimerize mature SP-B through interchain disulphide bond. Potential glycosilation site in the NH2-terminal propeptide (N129) is omitted as the protein is produced in bacteria. The second site (N311) in the COOH-terminal propeptide is also omitted. (DOC) [file pone.0158430.s001.doc]

**S1 Figure.**

**Scheme of human preproSP-B structure.**

**
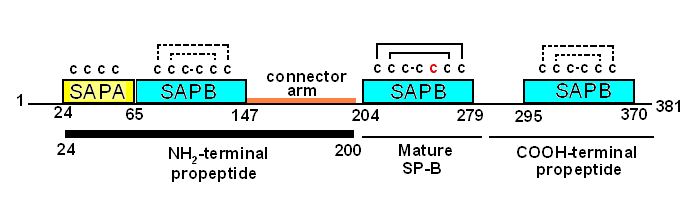
**

*Numbers* indicate the amino acid position at the beginning and end of the Saposin A-type like module (SAPA) or the B-type (SAPB). Signal peptide encompasses amino acids 1-23, NH_2_-terminal propeptide: 24-200 and mature SP-B: 201-279. Solid lines between cysteine residues indicate disulphide bonds and dashed lines indicate putative disulphide bonds by homology with bonds in saposin modules of preproSaposin. Red cys in mature SP-B serves to dimerize mature SP-B through interchain disulphide bond. Potential glycosilation site in the NH_2_-terminal propeptide (N^129^) is omitted as the protein is produced in bacteria. The second site (N^311^) in the COOH-terminal propeptide is also omitted.
