# Supplementary material for: Conformational Stability of the NH2-Terminal Propeptide of the Precursor of Pulmonary Surfactant Protein SP-B
Source: PLoS One. 2016 Jul 5;11(7):e0158430. doi: 10.1371/journal.pone.0158430 (PMC4933373; doi:10.1371/journal.pone.0158430)
Supplement: S2 Fig — Disordered regions in SP-BN have been predicted by Spritz v0.1 [1]. The total % disorder is 25.42, comprising three patches of 4, 22 and 19 amino acids respectively as is shown in the propeptide sequence (D means disorder and O means globular). The bigger patches correspond to connector arm. [1] Vullo A, Bortolami O, Pollastri G, Tosato SC, Spritz A: a server for the prediction of intrinsically disordered regions in protein sequences using kernel machines. Nucleic Acids Res. 2006;34: 164–168. (DOC) [file pone.0158430.s002.doc]

**S2 Figure.**

**Prediction of disordered regions in SP-B_N_**

Disordered regions in SP-B_N_ have been predicted by Spritz v0.1 [1]. The total % disorder is 25.42, comprising three patches of 4, 22 and 19 amino acids respectively as is shown in the propeptide sequence (D means disorder and O means globular). The bigger patches correspond to connector arm.

**24 27**

**AWTTSSLACAQGPEFWCQSLEQALQCRALGHCLQEVWGHVGADDLCQECEDIVHILNKMAKEAIFQDTMR**

DDDDOOOOOOOOOOOOOOOOOOOOOOOOOOOOOOOOOOOOOOOOOOOOOOOOOOOOOOOOOOOOOOOOOO
 **146**

**KFLEQECNVLPLKLLMPQCNQVLDDYFPLVIDYFQNQTDSNGICMHLGLCKSRQPEPEQEPGMSDPLPKP**

OOOOOOOOOOOOOOOOOOOOOOOOOOOOOOOOOOOOOOOOOOOOOOOOOOOODDDDDDDDDDDDDDDDDD
 **167 182 200**

**LRDPLPDPLLDKLVLPVLPGALQARPGPHTQDLSEQQ**

DDDDOOOOOOOOOOOOOODDDDDDDDDDDDDDDDDDD

# [1] A. Vullo, O. Bortolami, G. Pollastri, S.C. Tosato, Spritz: a server for the prediction of intrinsically disordered regions in protein sequences using kernel machines, Nucleic Acids Res. 34 (2006) 164-168.
