# Supplementary material for: Conformational Stability of the NH2-Terminal Propeptide of the Precursor of Pulmonary Surfactant Protein SP-B
Source: PLoS One. 2016 Jul 5;11(7):e0158430. doi: 10.1371/journal.pone.0158430 (PMC4933373; doi:10.1371/journal.pone.0158430)
Supplement: S3 Fig — The propeptide was incubated with 0.08% (v/v) glutaraldehyde (+GA) for 20 s and 120 s or without it (-GA) at 20°C (control sample, ~ 20 kDa). The positive control was 2 μg of the propeptide fused to the Maltose Binding Protein (F, ~ 62 kDa). To confirm and clarify the SP-BN oligomers, we carried out experiments with the bifunctional cross-linking agent glutaraldehyde. SP-BN samples (0.26 mg·mL-1) in 20 mM Tris-HCl, 500 mM NaCl pH 7.0 were incubated at 20°C with 0.08% (v/v) glutaraldehyde (GA, Amersham) during different times. This is a protein concentration above the concentration employed when only intramolecular crosslinking is desired (~ 0.1mg·mL-1) [1]. The reaction was finished by addition of 5 μL of loading buffer (5x; SDS, reducing conditions) to 20 μL of the sample and 5.2 μg of protein was applied to 12% SDS-PAGE. SP-BN was detected by immunoblotting with a primary monoclonal anti-proSP-B antibody (a gift of Dr Weaver, University of Cincinnati, USA) and bound antibody was probed with a secondary anti-Mouse IgG peroxidase-conjugate antibody (Sigma) as described [2]. The recombinant SP-BN in absence of GA shows a band of ~ 20 kDa corresponding to the monomer and a trace band migrating as the positive control (fusion protein) which is seen due to deliberate over-exposition. Incubation of SP-BN with 0.08% (v/v) GA for 20 s and 120 s decreased progressively the ~ 20 kDa band signal whereas new band signals were detected Those bands were assigned to putative dimer and trimer cross-linked forms of SP-BN respectively. In samples exposed to higher GA concentrations or incubated longer times at the same concentration, SP-BN was cross-linked to produce higher molecular mass species which either did not enter or remained at the non-lineal part of the gel (not shown). Attempts of detecting any SP-BN cross-linked species staining the gel with Coomassie, failed due to the small quantities of oligomers entering the gel. By the other hand, lower GA concentrations than 0.08% [file pone.0158430.s003.doc]

**Figure S3.**

**Glutaraldehyde cross-linking of SP-B_N_**

To confirm and clarify the SP-B_N_ oligomers, we carried out experiments with the bifunctional cross-linking agent glutaraldehyde. SP-B_N_ samples (0.26 mg·mL^-1^) in 20 mM Tris-HCl, 500 mM NaCl pH 7.0 were incubated at 20 ºC with 0.08 % (v/v) glutaraldehyde (GA, Amersham) during different times. This is a protein concentration above the concentration employed when only intramolecular crosslinking is desired (~ 0.1mg·mL^-1^) [2]. The reaction was finished by addition of 5 μL of loading buffer (5x; SDS, reducing conditions) to 20 μL of the sample and 5.2 μg of protein was applied to 12 % SDS-PAGE. SP-B_N_ was detected by immunoblotting with a primary monoclonal anti-proSP-B antibody (a gift of Dr Weaver, University of Cincinnati, USA) and bound antibody was probed with a secondary anti-Mouse IgG peroxidase-conjugate antibody (Sigma) as described [3].

**Time progress of SP-B_N_ cross-linked oligomers formation.** The propeptide was incubated with 0.08 % (v/v) glutaraldehyde (+GA) for 20 s and 120 s or without it (-GA) at 20 ºC (control sample, ~ 20 kDa). The positive control was 2 μg of the propeptide fused to the Maltose Binding Protein (F,~ 62 kDa).

Glutaraldehyde (GA) reacts with the amino group of the Lys side chains and, therefore has been used extensively to cross-link oligomeric proteins [4]. The recombinant SP-B_N_ in absence of GA shows a band of ~ 20 kDa corresponding to the monomer and a trace band migrating as the positive control (fusion protein) which is seen due to deliberate over-exposition. Incubation of SP-B_N_ with 0.08 % (v/v) GA for 20 s and 120 s decreased progressively the ~ 20 kDa band signal whereas new band signals were detected Those bands were assigned to putative dimer and trimer cross-linked forms of SP-B_N_ respectively. In samples exposed to higher GA concentrations or incubated longer times at the same concentration, SP-B_N_ was cross-linked to produce higher molecular mass species which either did not enter or remained at the non-lineal part of the gel (not shown). Attempts of detecting any SP-B_N_ cross-linked species staining the gel with Coomassie, failed due to the small quantities of oligomers entering the gel. By the other hand, lower GA concentrations than 0.08 % (v/v) were not effective to crosslink the protein.

[2] H. Ohno, F. Kurusu, Cytochrome C cross-linked with glutaraldehyde. Electrochemical response in poly(ethylene oxide) oligomers, Chem. Lett. 8 (1996) 693-694.

[3] A. Palacios, B. González, S. Alonso, J. Pérez-Gil, P. Estrada, Production of a recombinant form of the propeptide NH_2_-Terminal of the precursor of pulmonary surfactant protein B, Enzyme Microb. Technol. 40 (2006) 85-92.

[4] W.S. Craig, Determination of quaternary structure of an active enzyme using chemical cross-linking with glutaraldehyde, in S. Fleischer, B. Fleischer (Eds), Methods in Enzymology, New York, 156, 1988, pp. 335-345.
